# Crystal structures and circular dichroism of {2,2′-[(1*S*,2*S*)-1,2-di­phenyl­ethane-1,2-diylbis(nitrilophenyl­methanylyl­idene)]diphenolato}nickel(II) and its ethanol solvate

**DOI:** 10.1107/S2056989024010508

**Published:** 2024-11-08

**Authors:** Masataka Ito, Noriko Chikaraishi Kasuga, Ryo Matsuse, Masakazu Hirotsu

**Affiliations:** a Department of Chemistry, Faculty of Science, Kanagawa University, Kanagawa-ku, Yokohama 221-8686, Japan; University of Hyogo, Japan

**Keywords:** crystal structure, nickel(II) complex, tetra­dentate Schiff base ligand, phenyl group, circular dichroism

## Abstract

The title compound, [Ni(C_40_H_30_N_2_O_2_)] (**1**), with an optically active Schiff base ligand derived from 2-hy­droxy­benzo­phenone and (1*S*,2*S*)-1,2-di­phenyl­ethyl­enedi­amine, was crystallized as the solvent-free and ethanol solvate forms (**1** and **1**·2C_2_H_5_OH). In both structures, the two phenyl groups on the stereogenic centers of the *O*,*N*,*N*,*O*-tetra­dentate ligand are axially oriented, and the conformation of the central di­amine chelate ring is λ. The circular dichroism (CD) spectra of **1** and the analogous nickel(II) complex [Ni(C_30_H_26_N_2_O_2_)] (**2**) in solution show partially similar patterns in the 350–450 nm range, but are mirror images in the longer wavelength region (450–650 nm). In the latter region, the sign of CD for these complexes is sensitive to the substituents on the C=N carbon atoms (phenyl for **1** and methyl for **2**) rather than the di­amine chelate ring conformation.

## Abstract 2

A chiral nickel(II) Schiff base complex derived from 2-hy­droxy­benzo­phenone and (1*S*,2*S*)-1,2-di­phenyl­ethyl­enedi­amine shows a λ conformation of the central di­amine chelate ring. The substituents on the C=N carbon atoms significantly affect the circular dichroism spectra.

## 1. Chemical context

Metal complexes of chiral salen-type ligands derived from salicyl­aldehydes and di­amines have been employed as catalysts for asymmetric reactions in both homogeneous and heterogeneous systems (Canali & Sherrington, 1999 ▸; Cozzi, 2004 ▸; Zulauf *et al.*, 2010 ▸; Shaw & White, 2019 ▸; Abd El Sater *et al.*, 2019 ▸). In the chiral metallosalen complexes, the stereogenic centers are introduced to the *N*,*N*-chelate moiety of the *O*,*N*,*N*,*O*-tetra­dentate ligand by using chiral di­amines such as 1,2-cyclo­hexa­nedi­amine and 1,2-di­phenyl­ethyl­enedi­amine. It has been well established that the introduction of appropriate substituents at 3- and 5-positions of the salicyl­aldehyde effectively enhances the enanti­oselectivity (Nakajima *et al.*, 1990 ▸; Zhang *et al.*, 1990 ▸; Irie *et al.*, 1990 ▸; Ito & Katsuki, 1999 ▸). A modification of the C=N moiety can be achieved by the use of 2-hy­droxy­benzo­phenone, 3,5-di-*tert*-butyl-2-hy­droxy­aceto­phenone, or 3,5-di-*tert*-butyl-2-hy­droxy­valero­phenone in place of salicyl­aldehyde, and the catalytic properties of the C=N-modified complexes have been reported (Belokon *et al.*, 2004 ▸; Shaw & White, 2015 ▸). In these catalytic reactions, the conformation of the tetra­dentate ligands, which is imposed by the *N*,*N*-chelate moiety, plays an essential role in determining the stereoselectivity; therefore, elucidation of the solution structures is required.

**Figure 1:**
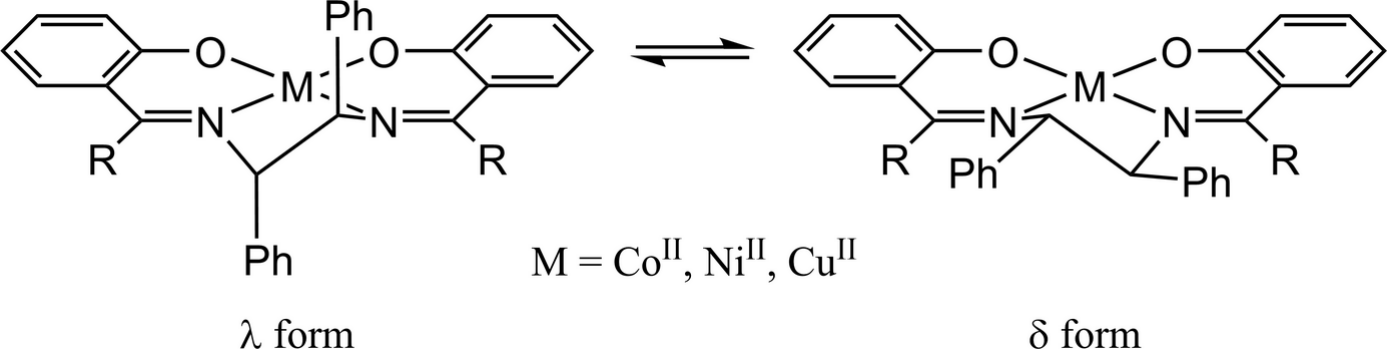
Conformers of tetra­dentate Schiff base complexes.

The circular dichroism (CD) spectra of the chiral salen-type metal complexes provide useful information on the solution structures in relation to the absolute configuration of the di­amines (Bosnich, 1968 ▸; Downing & Urbach, 1969 ▸, 1970 ▸; Pasini *et al.*, 1977 ▸). The λ and δ gauche conformations of the *N*,*N*-chelate ring derived from (1*S*,2*S*)-1,2-di­phenyl­ethyl­enedi­amine are inter­convertible in solution (Fig. 1 ▸). In the four-coordinate salen-type copper(II) complexes, the exciton couplet is observed in the 350 nm region, and the λ conformation of the Cu–N–C–C–N chelate ring is reflected by the negative–positive (lower to higher energy) exciton couplet (Downing & Urbach, 1969 ▸; Pasini *et al.*, 1977 ▸). The exciton couplet in this region, however, is not clear in analogous nickel(II) complexes, which is probably due to the overlapping of some other bands or the higher planarity (Downing & Urbach, 1970 ▸; Pasini *et al.*, 1977 ▸). Therefore, the substituent effect of the salen-type complexes on the CD spectra must be carefully investigated in order to discuss the solution structures.
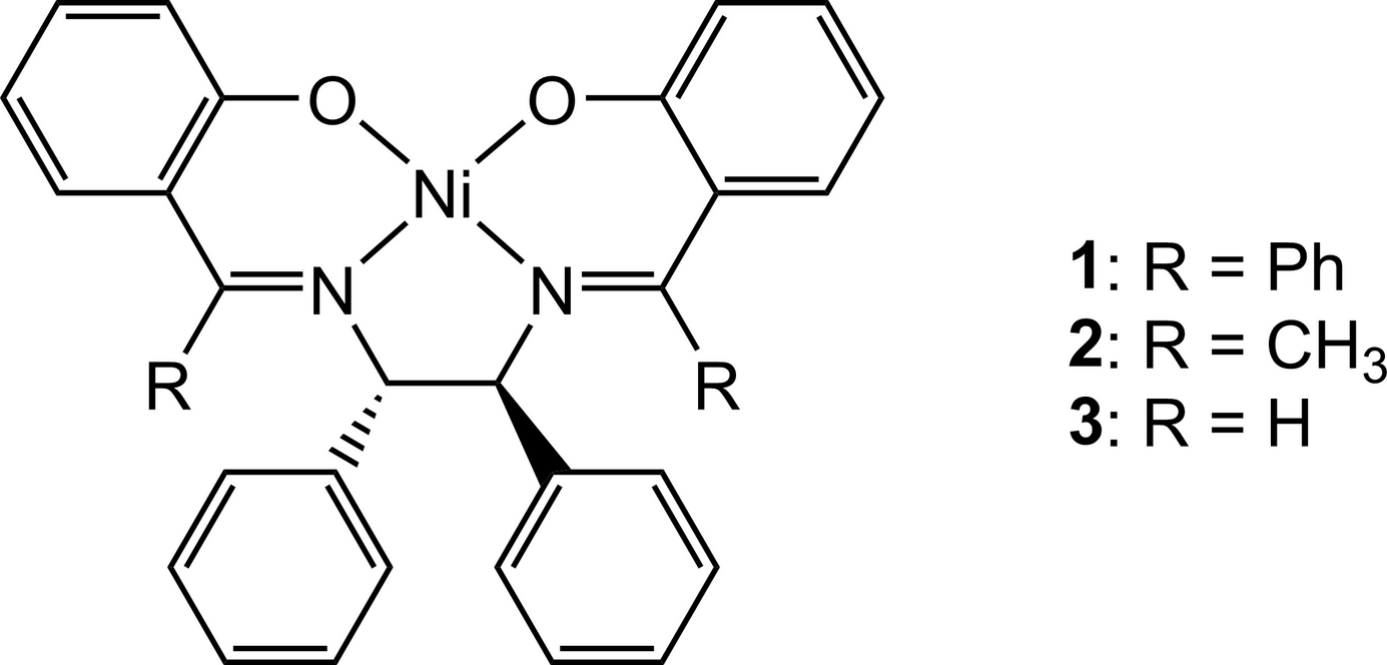


In this study we synthesized an optically active nickel(II) complex, [Ni(C_40_H_30_N_2_O_2_)] (**1**), in which the *O*,*N*,*N*,*O*-tetra­dentate ligand is derived from 2-hy­droxy­benzo­phenone and (1*S*,2*S*)-1,2-di­phenyl­ethyl­enedi­amine. The crystal structures of complex **1** and its ethanol solvate (**1**·2C_2_H_5_OH) are discussed in terms of the *N*,*N*-chelate ring conformation. Furthermore, the influence of the substituents on the C=N carbon atoms on the CD spectra in solution was investigated by the comparison with the analogous nickel(II) complex [Ni(C_30_H_26_N_2_O_2_)] (**2**) derived from 2′-hy­droxy­aceto­phenone and (1*S*,2*S*)-1,2-di­phenyl­ethyl­enedi­amine.

## 2. Structural commentary

**Figure 2:**
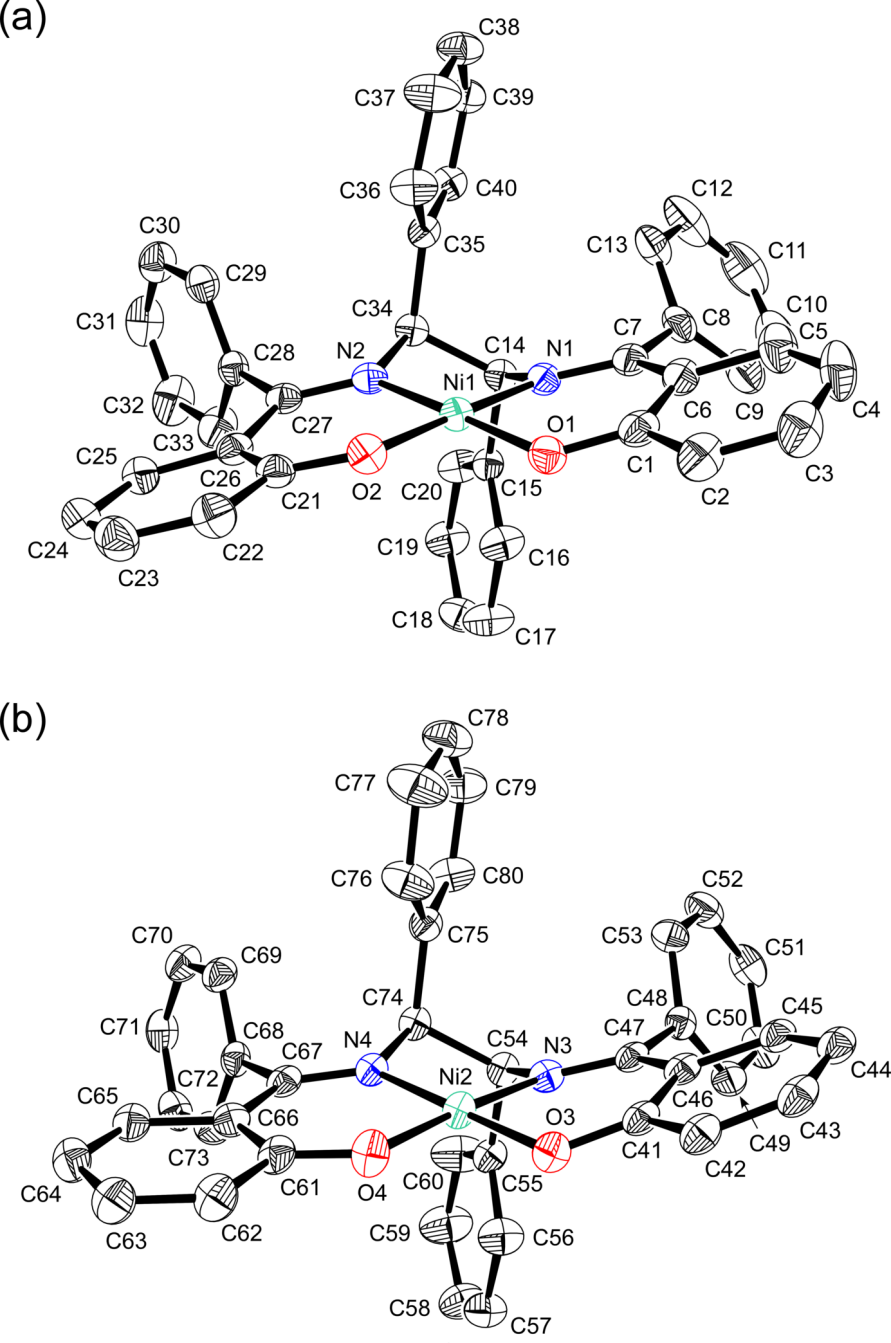
Perspective view of (*a*) mol­ecule *A* and (*b*) mol­ecule *B* in **1** with displacement ellipsoids at the 50% probability level. Hydrogen atoms are omitted for clarity.

**Figure 3:**
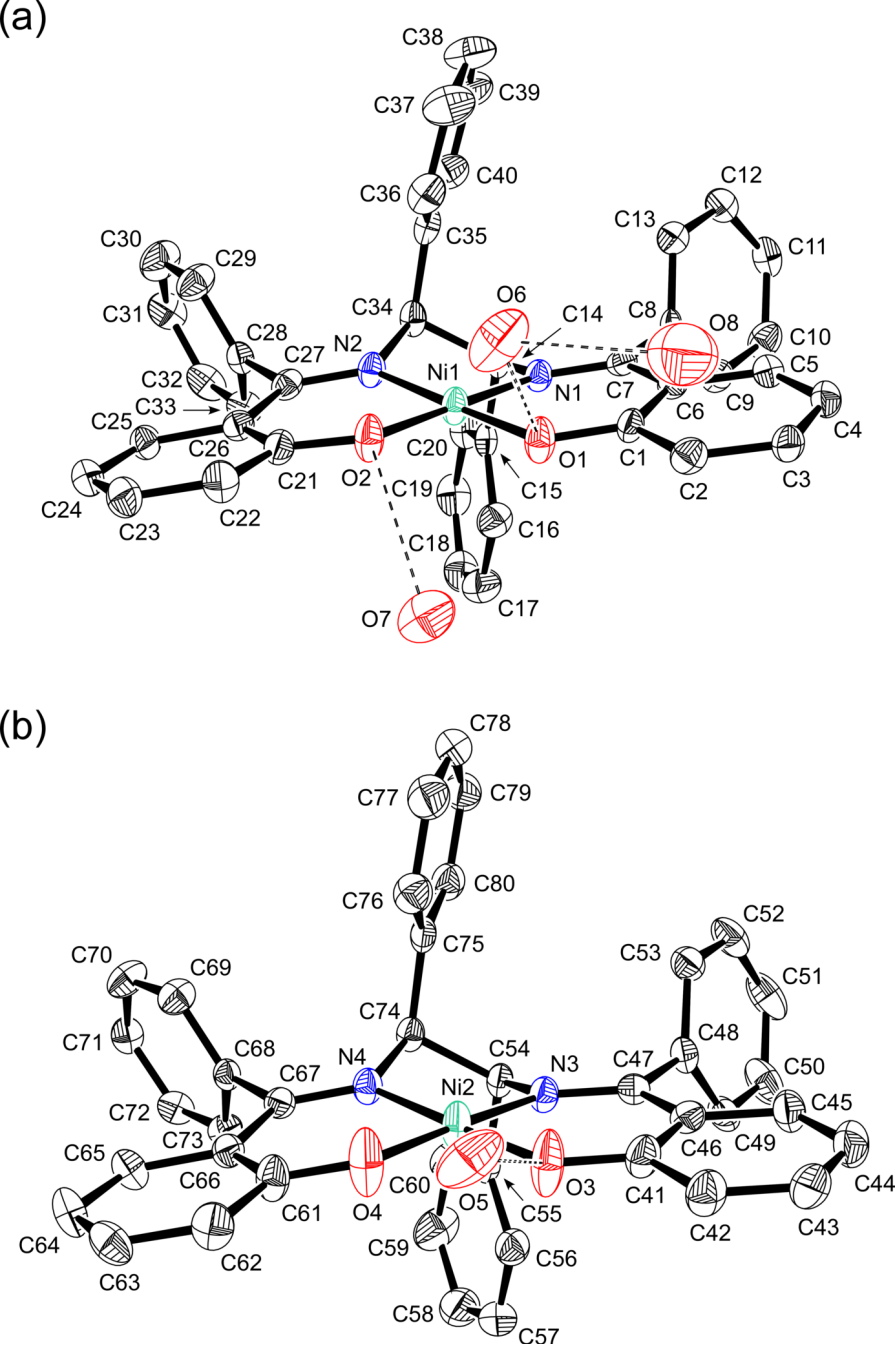
Perspective view of (*a*) mol­ecule *A*′ and (*b*) mol­ecule *B*′ in the ethanol solvate of **1** with displacement ellipsoids at the 50% probability level. Hydrogen atoms and ethyl groups of the ethanol mol­ecules are omitted for clarity. Hydrogen bonds are shown as dashed lines.

The solvent-free and ethanol solvate forms of complex **1** were obtained by changing the crystallization conditions: they crystallize in the non-centrosymmetric space groups *P*2_1_ and *P*1, respectively. The absolute structure was chosen based on the *S*,*S* configuration of the optically pure di­amine used and confirmed by the refined Flack parameters. Both forms contain two independent mol­ecules of **1** in the asymmetric unit, which are depicted as mol­ecules *A* and *A*′ (containing Ni1) and *B* and *B*′ (containing Ni2) in Figs. 2 ▸ and 3 ▸. Complex **1** consists of a Ni^2+^ ion and a dianionic *O*,*N*,*N*,*O*-tetra­dentate ligand, giving a pseudo-*C*_2_-symmetric square-planar geometry. The Ni atom sits in the N_2_O_2_ plane and is incorporated into two six-membered *O*,*N*-chelate rings and a five-membered *N*,*N*-chelate ring (Figs. 2 ▸ and 3 ▸). In the ethanol solvate (**1**·2C_2_H_5_OH, *Z* = 2), three of the four ethanol mol­ecules are bound to the phenolate O atoms through a hydrogen bond: two for the Ni1 site and one for the Ni2 site (Fig. 3 ▸).

The geometrical parameters around Ni for the solvent-free and ethanol solvate forms suggest that these hydrogen bonds do not affect the mol­ecular structures. The Ni—N and Ni—O bond distances are each within a small range for the four independent structures in these crystals (Tables 1 ▸ and 2 ▸). The four donor atoms show a slight tetra­hedral distortion: the root-mean-square deviations of the N_2_O_2_ plane are 0.0033 Å for mol­ecule *A* and 0.0275 Å for mol­ecule *B* in the solvent-free form, and 0.0163 Å for mol­ecule *A*′ and 0.0301 Å for mol­ecule *B*′ in the ethanol solvate form. These deviations are much smaller than those observed in the corresponding cobalt(II) and copper(II) complexes (0.10 Å, 0.20 Å, respectively; (Hirotsu *et al.*, 1996 ▸, 2009 ▸). The distortion from planarity of the ligand is caused by the conformation of the *N*,*N*-chelate ring. In these complexes, the two phenyl groups on the di­amine chelate are oriented axially with respect to the plane of the Schiff base ligand, which is due to the severe steric repulsion with the phenyl groups on the C=N carbon atoms (Hirotsu *et al.*, 1996 ▸). Consequently, the *S*,*S* configuration of the di­amine moiety leads to the λ gauche conformation of the *N*,*N*-chelate ring. The N—C—C—N torsion angles of **1** are in the range of −46.9 (3) to −49.6 (3)° (Tables 1 ▸ and 2 ▸), which are similar to those of the corresponding cobalt(II) and copper(II) complexes: Co, −45.1 (4)°; Cu, −51.70 (19)° (Hirotsu *et al.*, 1996 ▸, 2009 ▸).

**Figure 4:**
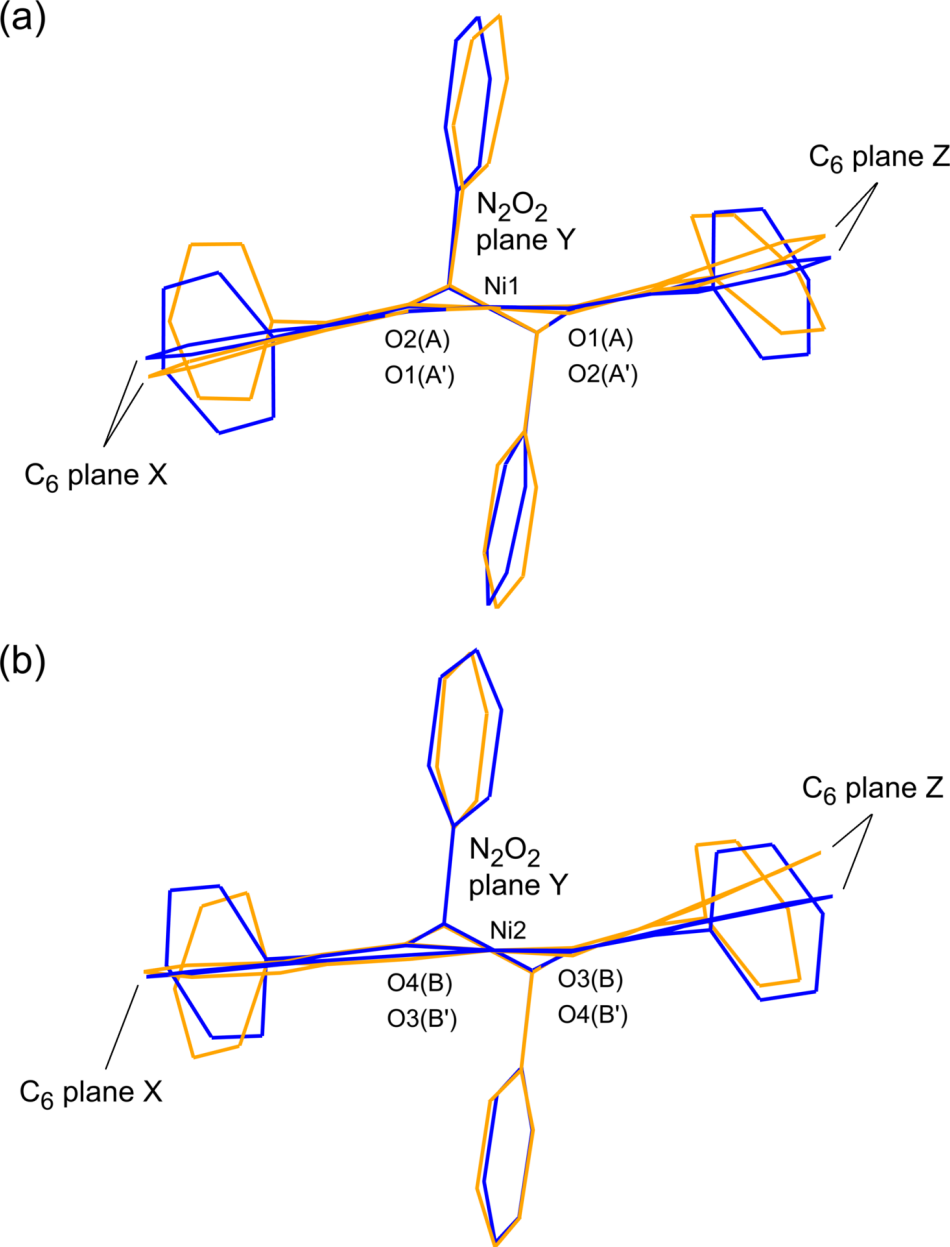
Overlays of the structures of (*a*) mol­ecules *A* (orange) and *A*′ (blue) and (*b*) mol­ecules *B* (orange) and *B*′ (blue).

Overlaying mol­ecules *A*, *A*′, *B*, and *B*′ revealed two types of bent conformations of the salen skeleton. Mol­ecules *A* and *A*′ adopt a stepped conformation, while mol­ecules *B* and *B*′ have an L-shaped conformation (Fig. 4 ▸). These conformations are described by the dihedral angles between the least square planes of the C_6_ ring (X, Z in Fig. 4 ▸) and N_2_O_2_ moieties (Y in Fig. 4 ▸): the inter­planar angles are 16.0 (1)° (X-Y), 18.8 (1)° (Y-Z) for *A*; 10.6 (1)° (X-Y), 11.8 (1)° (Y-Z) for *A*′; 2.3 (2)° (X-Y), 23.4 (1)° (Y-Z) for *B*; 6.2 (2)° (X-Y), 11.9 (1)° (Y-Z) for *B*′. The sum of the inter­planar angles of *A* or *B* is larger than that of *A*′ or *B*′, respectively. Therefore, the nickel(II) complex in the solvent-free form is more distorted than that in the ethanol solvate form. This suggests that the ethanol mol­ecules reduce the inter­molecular inter­actions between the complex mol­ecules.

The orientation of the phenyl group originating from the 1,2-di­phenyl­ethyl­enedi­amine is affected by the substituents R on the C=N carbon atoms. The crystal structures of the analogous nickel(II) complexes **2** (R = Me) and **3** (R = H) have been reported (Wang *et al.*, 2006 ▸; Ding, 2013 ▸). The phenyl groups in **2** are axially disposed relative to the ligand plane, while the phenyl groups in **3** occupy equatorial positions. In complex **2**, the axial disposition of the phenyl groups would be caused by the steric repulsion with the R groups (R = Me), as observed for **1** (R = Ph) and the corresponding copper(II) complexes with (*R,R*/*S,S*)- or (*R,S*)-configurations (Hirotsu *et al.*, 2009 ▸). Inter­estingly, the analogues of **3**, which have substituents on the phenolate rings, occupy the axial as well as the equatorial positions in the solid state (Averseng *et al.*, 2000 ▸; Wu *et al.*, 2003 ▸). The planer structure with equatorial phenyl groups observed for **3** may be advantageous in terms of the effect of crystal packing.

In the structure of **1**, several C—H bonds of the axially disposed phenyl groups are close to Ni. The Ni⋯H distances are 2.72–2.96 Å for the solvent-free form and 2.66–2.91 Å for the ethanol solvate form. These structural features are indicative of anagostic inter­actions (Mitoraj *et al.*, 2019 ▸).

## 3. Supra­molecular features

**Figure 5:**
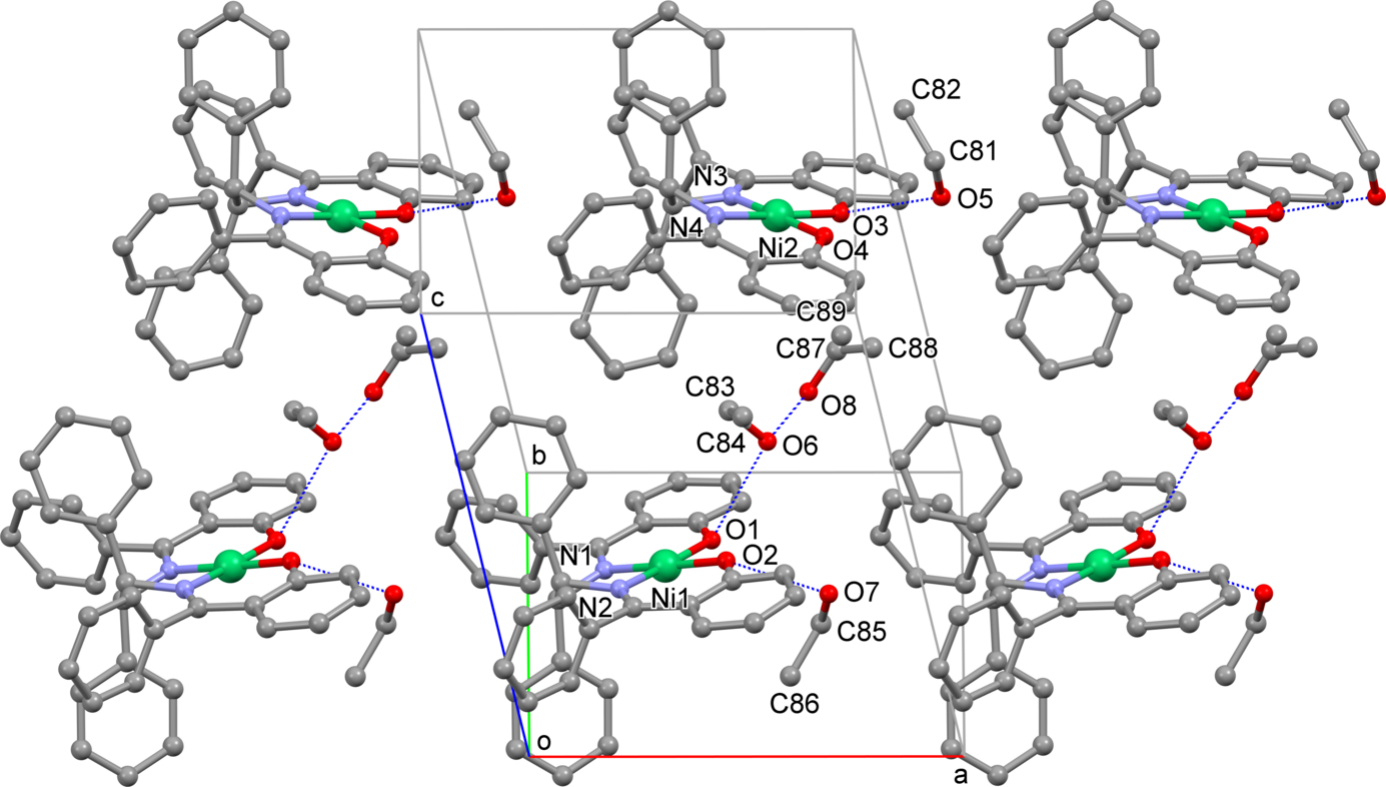
The crystal packing of the ethanol solvate of **1**. Hydrogen atoms are omitted for clarity. Hydrogen bonds are shown as blue dashed lines.

In the ethanol solvate, the pseudo-*C*_2_ axis of each complex mol­ecule is nearly parallel to the *a* axis of the crystal cell (Fig. 5 ▸). The space around the phenolate O donor atoms is occupied by ethanol mol­ecules. As mentioned above, the three ethanol mol­ecules are bound to the phenolate O atoms through a hydrogen bond. The remaining ethanol mol­ecule, which is disordered, occupies the space between the two complex mol­ecules and forms a hydrogen bond with the ethanol mol­ecule. Weak CH(phen­yl)⋯O(ethanol) inter­actions are observed between the asymmetric units (Table 4 ▸ ▸).

In the solvent-free form, there are short contacts such as CH(phen­yl)⋯O hydrogen bonds between the complex mol­ecules (Table 3 ▸). The torsion angles between N=C and the phenyl group (R) [61.6 (5)–102.4 (4)°] deviate largely from those of the ethanol solvate [79.2 (5)–85.4 (4)°] (Tables 1 ▸ and 2 ▸, Fig. 4 ▸). Unlike complex **3**, the conformational change of the *N*,*N*-chelate ring in **1** is not effective in forming the inter­molecular inter­actions while avoiding inter­molecular repulsion because of the intra­molecular repulsion between the phenyl groups.

## 4. Database survey

Several transition-metal complexes of the Schiff base derived from 2-hy­droxy­benzo­phenone and 1,2-di­phenyl­ethyl­enedi­amine, including (1*R*,2*R*)-, (1*S*,2*S*)-, and (1*R*,2*S*)-isomers have been crystallographically characterized. As mentioned above, the racemic cobalt(II) and copper(II) complexes show a similar square-planar geometry with tetra­hedral distortion (Hirotsu *et al.*, 1996 ▸; Hirotsu *et al.*, 2009 ▸). The meso copper(II) complex with (*R*,*S*)-configuration is also square-planar but less tetra­hedrally distorted (Hirotsu *et al.*, 2009 ▸). The chlorido manganese(III) complex [Mn(C_40_H_30_N_2_O_2_)Cl] has a square-pyramidal structure, in which the di­amine chelate moiety with the (*S*,*S*)-configuration gives a λ *gauche* conformation with axially disposed phenyl groups (Hirotsu *et al.*, 1995 ▸)

## 5. Circular dichroism

In the nickel(II) complexes of the *O*,*N*,*N*,*O*-Schiff base ligands derived from (1*S*,2*S*)-1,2-di­phenyl­ethyl­enedi­amine, the predominant conformation of the *N*,*N*-chelate ring is dependent on the R substituents (Fig. 1 ▸). For complex **3** (R = H), the δ conformation is found in the solid state if the di­amine chelate has the (*S*,*S*)-configuration (Ding, 2013 ▸). In solution, however, analysis of the CD spectra for a series of optically active Ni complexes suggests tentatively that complex **3** takes the λ conformation: although no exciton couplet is observed, **3** exhibits opposite behavior to the complex derived from (1*S*,2*S*)-1,2-cyclo­hexa­nedi­amine in the range 300–500 nm (Pasini *et al.*, 1977 ▸). In the case of complex **2** (R = Me), the solution structure is assigned to the λ conformation from the CD spectrum in methanol (Wang *et al.*, 2006 ▸).

For complex **1**, the ^1^H NMR spectrum (CDCl_3_) suggests free rotation of the phenyl groups on the *N*,*N*-chelate ring in solution, whereas restricted rotation of those on the C=N moieties. Furthermore, the methine proton signal of **1** (δ 4.05) appeared at a higher field than that of **2** (δ 4.73), due to the ring current effect of the additional phenyl groups. These findings are consistent with the λ conformation observed in the crystal structures.

**Figure 6:**
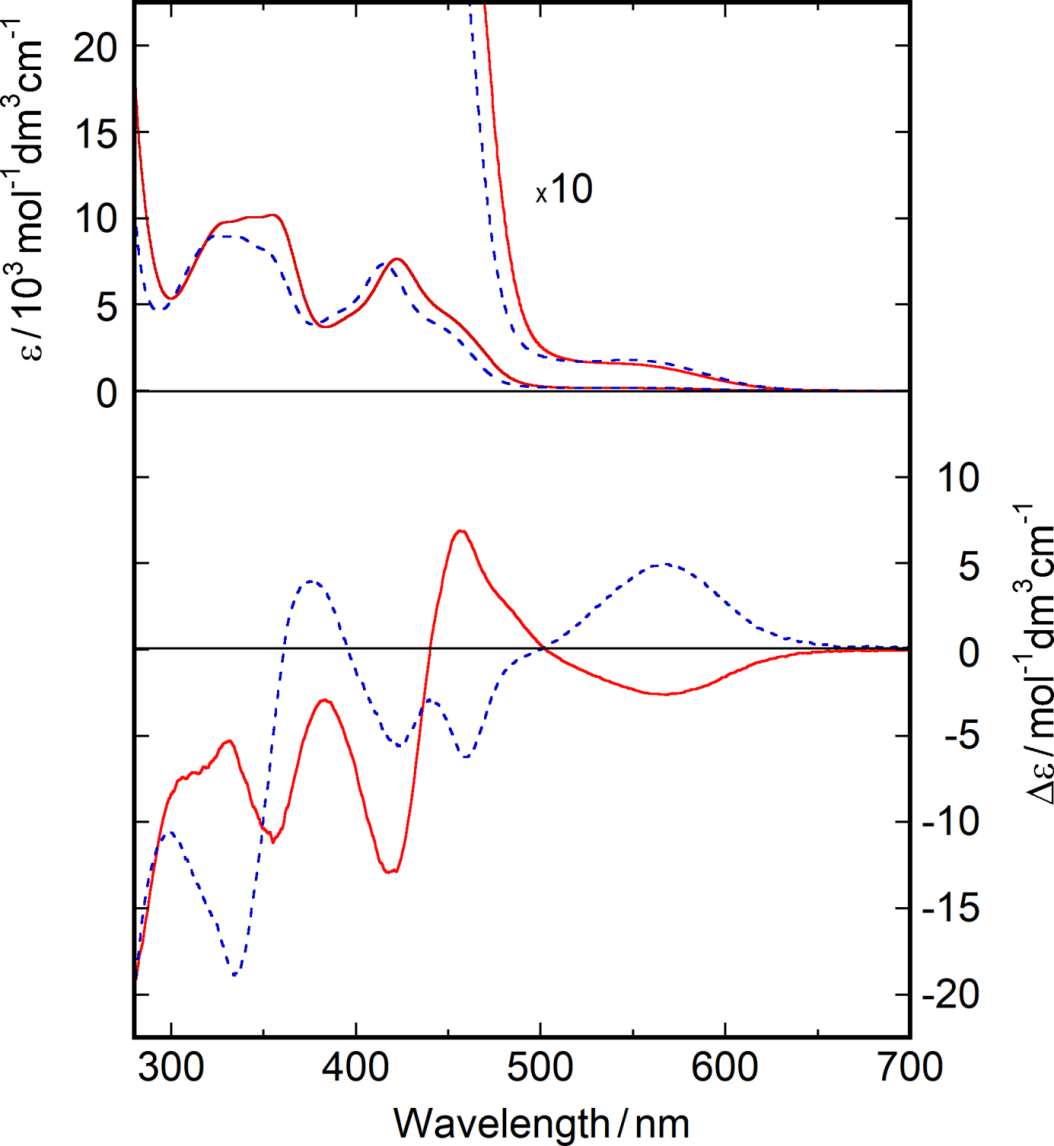
(Top) Electronic spectra of complexes **1** (red solid line) and **2** (blue dashed line) in di­chloro­methane. (Bottom) CD spectra of complexes **1** (red solid line) and **2** (blue dashed line) in di­chloro­methane.

To elucidate the effect of the R substituents on the CD spectral patterns, absorption and CD spectra of complexes **1** and **2** were measured in di­chloro­methane (Fig. 6 ▸). The intense absorption bands at 370–500 nm are due to charge-transfer transitions, including π–π* transitions of the azomethine chromophore, and a red-shift is observed for **1**. A weak shoulder at low energy is considered to originate from the *d*–*d* transitions (Downing & Urbach, 1970 ▸). In the CD spectra, a mirror image is observed in the range of 450–650 nm, but not in the higher energy region above 450 nm. Both **1** and **2** show a negative CD band at around 420 nm, suggesting that the preferred conformation is λ as expected from the crystal structures. Thus, in the salen-type nickel(II) complexes, the sign of CD in the 450–650 nm region is readily reversed when the R substituents on the C=N carbon atoms are different even if the conformation of the *N*,*N*-chelate ring is the same.

## 6. Synthesis and crystallization

**General Procedures.** NMR spectra were recorded on a JEOL ECZ-600 spectrometer at room temperature. Elemental analysis was performed by A Rabbit Science Co., Ltd. UV-vis spectra were measured on a JASCO V-770 spectrometer. Circular dichroism spectra were measured on a JASCO J-820 spectropolarimeter. Complex **2** was prepared according to a literature procedure (Wang *et al.*, 2006 ▸).

**[Ni(C_40_H_30_N_2_O_2_)] (1).** (1*S*,2*S*)-1,2-di­phenyl­ethyl­enedi­amine (0.42 g, 2.0 mmol) and 2-hy­droxy­benzo­phenone (0.79 g, 4.0 mmol) were refluxed in ethanol (10 mL) for 37 h. After cooling to room temperature, the resulting yellow precipitate was collected by filtration, washed with ethanol, and dried under reduced pressure to afford the Schiff base ligand (0.60 g, 52%). ^1^H NMR (600 MHz, CDCl_3_): δ 4.75 (*s*, 2H, N–CH–CH–N), 6.60 (*ddd*, *J* = 8.0, 7.0, 1.0 Hz, 2H), 6.66 (*dd*, *J* = 7.9, 1.6 Hz, 2H), 6.69 (*d*, *J* = 7.3 Hz, 2H), 6.76 (*d*, *J* = 6.9 Hz, 2H), 6.90–6.93 (*m*, 4H), 7.09–7.14 (*m*, 8H), 7.25–7.29 (*m*, 4H), 7.41 (*t*, *J* = 7.4 Hz, 2H), 7.45 (*t*, *J* = 7.4 Hz, 2H), 15.47 (*s*, 2H, OH). The ligand (115 mg, 0.20 mmol) and nickel(II) acetate tetra­hydrate (50 mg, 0.20 mmol) were suspended in ethanol (10 mL) and then refluxed for 3 h to give a red–brown suspension. The precipitate was collected by filtration, washed with ethanol, and dried under reduced pressure to yield complex **1** as a red–brown solid (106 mg, 82%). ^1^H NMR (600 MHz, CDCl_3_): δ 4.05 (*s*, 2H, N–CH–CH–N), 6.18 [*d*, *J* = 7.7 Hz, 2H, N=CPh(*o*)], 6.27 [*ddd*, *J* = 8.2, 6.2, 1.8 Hz, 2H, C(phenolato, 4)-H), 6.36 [*dd*, *J* = 8.2, 1.2 Hz, 2H, C(phenolato, 3)–H], 6.44 [*d*, *J* = 7.7 Hz, 2H, N=CPh(*o*)], 7.06 [*t*, *J* = 7.6 Hz, 2H, N=CPh(*m*)], 7.12–7.18 [*m*, 4H, C(phenolato, 5, 6)–H], 7.18 [*t*, *J* = 7.6 Hz, 2H, N=CPh(*m*)], 7.27 [*t*, *J* = 7.5 Hz, 2H, N=CPh(*p*)], 7.37 [*t*, *J* = 7.4 Hz, 2H, N–CPh(*p*)–CPh(*p*)–N], 7.44 [*t*, *J* = 7.5 Hz, 4H, N–CPh(*m*)–CPh(*m*)–N], 8.09 [*d*, *J* = 7.5 Hz, 4H, N–CPh(*o*)–CPh(*o*)–N]. Analysis calculated for C_40_H_30_N_2_NiO_2_·0.6H_2_O: C, 75.05; H, 4.91; N, 4.38. Found: C, 74.77; H, 4.58; N, 4.55. The solid was recrystallized by slow evaporation of a di­chloro­methane/ethanol solution to yield single crystals of the ethanol solvate, which were suitable for X-ray diffraction analysis. The solvent-free form was obtained by slow evaporation from a di­chloro­methane/2-propanol solution.

## 7. Refinement

Crystal data, data collection, and structure refinement details are summarized in Table 5 ▸. All non-hydrogen atoms were refined anisotropically. In the ethanol solvate, one of the four ethanol mol­ecules was modeled as disordered over two positions at the terminal carbon atom, with occupancy factors refined to 0.64 (4) and 0.36 (4). Hydrogen atoms were placed in calculated positions with C—H(aromatic) = 0.95 Å, C—H(meth­yl) = 0.98 Å, C—H(methyl­ene) = 0.99 Å, C—H(methine) = 1.00 Å, and O—H = 0.84 Å, and refined using a riding model with *U*_iso_(H) = 1.2*U*_eq_(C), 1.5*U*_eq_(C), 1.2*U*_eq_(C), 1.2*U*_eq_(C), and 1.5*U*_eq_(C), respectively.

## Supplementary Material

Crystal structure: contains datablock(s) 1, 1_EtOH. DOI: 10.1107/S2056989024010508/ox2008sup1.cif

Structure factors: contains datablock(s) 1. DOI: 10.1107/S2056989024010508/ox20081sup4.hkl

Structure factors: contains datablock(s) 1_EtOH. DOI: 10.1107/S2056989024010508/ox20081_EtOHsup5.hkl

CCDC references: 2394792, 2394791

Additional supporting information:  crystallographic information; 3D view; checkCIF report

## Figures and Tables

**Table 1:** Selected geometric parameters (Å, °) for **1**[Chem scheme1]

Ni1—O1	1.833 (2)	Ni2—O3	1.847 (2)
Ni1—O2	1.836 (3)	Ni2—O4	1.825 (3)
Ni1—N1	1.861 (3)	Ni2—N4	1.867 (3)
Ni1—N2	1.863 (3)	Ni2—N3	1.861 (3)
			
O1—Ni1—N1	93.37 (13)	O3—Ni2—N3	92.66 (12)
O2—Ni1—N2	93.97 (12)	O4—Ni2—N4	94.30 (12)
N1—Ni1—N2	87.42 (12)	N3—Ni2—N4	87.60 (13)
			
N1—C14—C34—N2	−49.6 (3)	N3—C54—C74—N4	−47.8 (3)
N1—C7—C8—C13	61.6 (5)	N3—C47—C48—C53	77.9 (4)
N2—C27—C28—C33	101.6 (4)	N4—C67—C68—C73	102.4 (4)

**Table 2:** Selected geometric parameters (Å, °) for **1**·2C_2_H_5_OH[Chem scheme1]

Ni1—O1	1.828 (3)	Ni2—O3	1.838 (3)
Ni1—O2	1.833 (3)	Ni2—O4	1.826 (3)
Ni1—N1	1.863 (3)	Ni2—N3	1.852 (3)
Ni1—N2	1.852 (3)	Ni2—N4	1.860 (3)
			
O1—Ni1—N1	94.47 (13)	O3—Ni2—N3	94.70 (13)
O2—Ni1—N2	94.11 (13)	O4—Ni2—N4	93.95 (13)
N2—Ni1—N1	87.42 (14)	N3—Ni2—N4	87.52 (14)
			
N1—C14—C34—N2	−47.4 (4)	N3—C54—C74—N4	−46.9 (3)
N1—C7—C8—C13	84.1 (4)	N3—C47—C48—C53	79.2 (5)
N2—C27—C28—C33	80.3 (5)	N4—C67—C68—C73	85.4 (4)

**Table 3:** Hydrogen-bond geometry (Å, °) for **1**[Chem scheme1]

*D*—H⋯*A*	*D*—H	H⋯*A*	*D*⋯*A*	*D*—H⋯*A*
C43—H43⋯O1^i^	0.95	2.60	3.500 (4)	159
C51—H51⋯O3^ii^	0.95	2.52	3.417 (5)	158
C71—H71⋯O1	0.95	2.63	3.308 (4)	128
C71—H71⋯O2	0.95	2.62	3.562 (4)	173

Symmetry codes: (i) 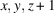; (ii) 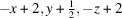.

**Table 4:** Hydrogen-bond geometry (Å, °) for **1**·2C_2_H_5_OH[Chem scheme1]

*D*—H⋯*A*	*D*—H	H⋯*A*	*D*⋯*A*	*D*—H⋯*A*
O5—H5*A*⋯O3	0.84	2.17	2.964 (5)	156
O6—H6⋯O1	0.84	2.13	2.928 (5)	158
O8—H8⋯O6	0.84	1.92	2.760 (6)	174
O7—H7⋯O2	0.84	2.16	2.988 (4)	170
C60—H60⋯O5^i^	0.95	2.38	3.312 (6)	166
C12—H12⋯O8^i^	0.95	2.44	3.388 (7)	175
C40—H40⋯O7^i^	0.95	2.48	3.328 (5)	148

Symmetry code: (i) 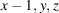.

**Table 5:** Experimental details

	**1**	**1**·2C_2_H_5_OH
Crystal data		
Chemical formula	[Ni(C_40_H_30_N_2_O_2_)]	[Ni(C_40_H_30_N_2_O_2_)]·2C_2_H_6_O
*M*_r_	629.37	721.50
Crystal system, space group	Monoclinic, *P*2_1_	Triclinic, *P*1
Temperature (K)	120	120
*a*, *b*, *c* (Å)	9.5487 (2), 17.8992 (3), 18.0001 (3)	10.5830 (2), 12.4110 (2), 13.9837 (2)
α, β, γ (°)	90, 94.103 (2), 90	94.352 (1), 100.599 (1), 90.584 (1)
*V* (Å^3^)	3068.59 (10)	1799.61 (5)
*Z*	4	2
Radiation type	Mo *K*α	Mo *K*α
μ (mm^−1^)	0.67	0.59
Crystal size (mm)	0.28 × 0.22 × 0.04	0.26 × 0.20 × 0.09

Data collection		
Diffractometer	Rigaku Oxford Diffraction, Synergy Custom system, HyPix	Rigaku Oxford Diffraction, Synergy Custom system, HyPix
Absorption correction	Multi-scan (*CrysAlis PRO*; Rigaku OD, 2024 ▸)	Multi-scan (*CrysAlis PRO*; Rigaku OD, 2018 ▸)
*T*_min_, *T*_max_	0.890, 1.000	0.824, 1.000
No. of measured, independent and observed [*I* > 2σ(*I*)] reflections	58868, 11251, 10665	24831, 13032, 12354
*R*_int_	0.051	0.025
(sin θ/λ)_max_ (Å^−1^)	0.602	0.602

Refinement		
*R*[*F*^2^ > 2σ(*F*^2^)], *wR*(*F*^2^), *S*	0.033, 0.084, 1.06	0.035, 0.083, 1.03
No. of reflections	11251	13032
No. of parameters	811	938
No. of restraints	1	9
H-atom treatment	H-atom parameters constrained	H-atom parameters constrained
Δρ_max_, Δρ_min_ (e Å^−3^)	0.48, −0.35	0.32, −0.37
Absolute structure	Flack *x* determined using 4774 quotients [(*I*^+^)−(*I*^−^)]/[(*I*^+^)+(*I*^−^)] (Parsons *et al.*, 2013 ▸)	Flack *x* determined using 5653 quotients [(*I*^+^)−(*I*^−^)]/[(*I*^+^)+(*I*^−^)] (Parsons *et al.*, 2013 ▸)
Absolute structure parameter	−0.009 (6)	−0.012 (5)

Computer programs: *CrysAlis PRO* (Rigaku OD, 2018 ▸, 2024 ▸), *SHELXT2018/2* (Sheldrick, 2015*a* ▸), *SHELXL2018/3* (Sheldrick, 2015*b* ▸), *OLEX2* (Dolomanov *et al.*, 2009 ▸), *ORTEP-3 for Windows* (Farrugia, 2012 ▸) and *Mercury* (Macrae *et al.*, 2020 ▸).

## supplementary crystallographic information

### {2,2'-[(1S,2S)-1,2-Diphenylethane-1,2-diylbis(nitrilophenylmethanylylidene)]diphenolato}nickel(II) (1) Crystal data

**Table:**

[Ni(C_40_H_30_N_2_O_2_)]	*F*(000) = 1312
*M**_r_* = 629.37	*D*_x_ = 1.36 Mg m^−^^3^
Monoclinic, *P*2_1_	Mo *K*α radiation, λ = 0.71073 Å
*a* = 9.5487 (2) Å	Cell parameters from 28038 reflections
*b* = 17.8992 (3) Å	θ = 3.0–31.0°
*c* = 18.0001 (3) Å	µ = 0.67 mm^−^^1^
β = 94.103 (2)°	*T* = 120 K
*V* = 3068.59 (10) Å^3^	Platelet, orange
*Z* = 4	0.28 × 0.22 × 0.04 mm

### {2,2'-[(1S,2S)-1,2-Diphenylethane-1,2-diylbis(nitrilophenylmethanylylidene)]diphenolato}nickel(II) (1) Data collection

**Table:**

Rigaku Oxford Diffraction, Synergy Custom system, HyPix diffractometer	11251 independent reflections
Radiation source: Rotating-anode X-ray tube, Rigaku (Mo) X-ray Source	10665 reflections with *I* > 2σ(*I*)
Mirror monochromator	*R*_int_ = 0.051
Detector resolution: 10.0000 pixels mm^-1^	θ_max_ = 25.4°, θ_min_ = 3.0°
ω scans	*h* = −11→11
Absorption correction: multi-scan (CrysAlisPro; Rigaku OD, 2024)	*k* = −21→21
*T*_min_ = 0.890, *T*_max_ = 1.000	*l* = −21→21
58868 measured reflections	

### {2,2'-[(1S,2S)-1,2-Diphenylethane-1,2-diylbis(nitrilophenylmethanylylidene)]diphenolato}nickel(II) (1) Refinement

**Table:**

Refinement on *F*^2^	Hydrogen site location: inferred from neighbouring sites
Least-squares matrix: full	H-atom parameters constrained
*R*[*F*^2^ > 2σ(*F*^2^)] = 0.033	*w* = 1/[σ^2^(*F*_o_^2^) + (0.0549*P*)^2^ + 0.2247*P*] where *P* = (*F*_o_^2^ + 2*F*_c_^2^)/3
*wR*(*F*^2^) = 0.084	(Δ/σ)_max_ = 0.001
*S* = 1.06	Δρ_max_ = 0.48 e Å^−^^3^
11251 reflections	Δρ_min_ = −0.35 e Å^−^^3^
811 parameters	Absolute structure: Flack *x* determined using 4774 quotients [(*I*^+^)-(*I*^-^)]/[(*I*^+^)+(*I*^-^)] (Parsons *et al.*, 2013)
1 restraint	Absolute structure parameter: −0.009 (6)
Primary atom site location: dual	

### {2,2'-[(1S,2S)-1,2-Diphenylethane-1,2-diylbis(nitrilophenylmethanylylidene)]diphenolato}nickel(II) (1) Special details

**Table:**

Geometry. All esds (except the esd in the dihedral angle between two l.s. planes) are estimated using the full covariance matrix. The cell esds are taken into account individually in the estimation of esds in distances, angles and torsion angles; correlations between esds in cell parameters are only used when they are defined by crystal symmetry. An approximate (isotropic) treatment of cell esds is used for estimating esds involving l.s. planes.

### {2,2'-[(1S,2S)-1,2-Diphenylethane-1,2-diylbis(nitrilophenylmethanylylidene)]diphenolato}nickel(II) (1) Fractional atomic coordinates and isotropic or equivalent isotropic displacement parameters (Å^2^)

**Table:**

	*x*	*y*	*z*	*U*_iso_*/*U*_eq_	
Ni1	0.49842 (4)	0.49807 (2)	0.37750 (2)	0.02359 (11)	
Ni2	0.70992 (5)	0.43794 (2)	0.96494 (2)	0.02393 (11)	
O1	0.6338 (3)	0.43286 (15)	0.41469 (13)	0.0290 (5)	
O3	0.7515 (3)	0.41402 (13)	1.06389 (13)	0.0284 (5)	
O4	0.6209 (3)	0.34771 (15)	0.95874 (14)	0.0325 (6)	
N1	0.3703 (3)	0.42227 (16)	0.35244 (16)	0.0251 (7)	
O2	0.6272 (3)	0.57118 (14)	0.40411 (14)	0.0294 (6)	
N4	0.6659 (3)	0.46219 (15)	0.86511 (15)	0.0230 (6)	
N3	0.8105 (3)	0.52698 (16)	0.97331 (16)	0.0241 (6)	
N2	0.3612 (3)	0.56557 (17)	0.34175 (16)	0.0242 (6)	
C48	0.9121 (4)	0.64063 (19)	1.02533 (18)	0.0241 (7)	
C21	0.6011 (4)	0.6425 (2)	0.4124 (2)	0.0280 (8)	
C28	0.2295 (4)	0.68120 (19)	0.3231 (2)	0.0259 (8)	
C67	0.6076 (4)	0.42011 (19)	0.81279 (19)	0.0250 (7)	
C74	0.7081 (4)	0.53926 (19)	0.84882 (19)	0.0245 (7)	
H74	0.729469	0.542734	0.795349	0.029*	
C47	0.8456 (3)	0.56564 (19)	1.03267 (18)	0.0229 (7)	
C43	0.7816 (4)	0.4867 (2)	1.25053 (19)	0.0302 (8)	
H43	0.766789	0.469454	1.299272	0.036*	
C14	0.2269 (4)	0.45374 (18)	0.34426 (18)	0.0246 (7)	
H14	0.163361	0.418802	0.314485	0.030*	
C7	0.3957 (4)	0.3511 (2)	0.3419 (2)	0.0271 (8)	
C29	0.2159 (4)	0.7029 (2)	0.2492 (2)	0.0300 (8)	
H29	0.285340	0.689186	0.216544	0.036*	
C68	0.6060 (4)	0.4465 (2)	0.73284 (18)	0.0256 (7)	
C46	0.8208 (3)	0.53767 (19)	1.10636 (19)	0.0235 (7)	
C63	0.4332 (4)	0.2070 (2)	0.8513 (2)	0.0367 (9)	
H63	0.396323	0.158603	0.859260	0.044*	
C57	1.1432 (5)	0.4161 (2)	0.8683 (2)	0.0392 (9)	
H57	1.184525	0.372500	0.890556	0.047*	
C58	1.1931 (4)	0.4442 (3)	0.8055 (2)	0.0408 (9)	
H58	1.274262	0.423094	0.786213	0.049*	
C78	0.3875 (5)	0.7025 (3)	0.8871 (3)	0.0457 (11)	
H78	0.318822	0.739063	0.896309	0.055*	
C75	0.5930 (4)	0.5952 (2)	0.86369 (19)	0.0268 (7)	
C54	0.8436 (4)	0.55343 (19)	0.89849 (18)	0.0233 (7)	
H54	0.863197	0.608293	0.900571	0.028*	
C41	0.7777 (3)	0.46268 (19)	1.11764 (19)	0.0245 (7)	
C39	0.1681 (4)	0.4656 (2)	0.1013 (2)	0.0308 (8)	
H39	0.089236	0.448033	0.071073	0.037*	
C22	0.7125 (4)	0.6881 (2)	0.4423 (2)	0.0353 (9)	
H22	0.801998	0.666249	0.454270	0.042*	
C42	0.7620 (4)	0.4387 (2)	1.19146 (18)	0.0291 (7)	
H42	0.737296	0.388169	1.200234	0.035*	
C61	0.5601 (4)	0.3158 (2)	0.8999 (2)	0.0285 (8)	
C69	0.5173 (4)	0.5028 (2)	0.70400 (19)	0.0317 (8)	
H69	0.449771	0.524300	0.733838	0.038*	
C24	0.5635 (5)	0.7967 (2)	0.4387 (2)	0.0372 (9)	
H24	0.550658	0.848194	0.448786	0.045*	
C35	0.2667 (3)	0.51123 (18)	0.21998 (18)	0.0236 (7)	
C53	0.8279 (4)	0.7015 (2)	1.0041 (2)	0.0305 (8)	
H53	0.728710	0.696194	0.999718	0.037*	
C62	0.5015 (4)	0.2442 (2)	0.9094 (2)	0.0350 (9)	
H62	0.509713	0.221457	0.957164	0.042*	
C23	0.6942 (4)	0.7628 (2)	0.4544 (2)	0.0393 (9)	
H23	0.771370	0.792081	0.473700	0.047*	
C55	0.9678 (4)	0.51311 (19)	0.86888 (19)	0.0264 (8)	
C25	0.4541 (4)	0.7542 (2)	0.4084 (2)	0.0316 (8)	
H25	0.365185	0.777108	0.397660	0.038*	
C40	0.1533 (4)	0.4856 (2)	0.1743 (2)	0.0290 (8)	
H40	0.063786	0.481666	0.193838	0.035*	
C1	0.6418 (4)	0.3616 (2)	0.3995 (2)	0.0302 (8)	
C72	0.7107 (4)	0.4394 (2)	0.61553 (19)	0.0342 (8)	
H72	0.776265	0.417089	0.584985	0.041*	
C34	0.2420 (4)	0.52701 (19)	0.30101 (19)	0.0242 (7)	
H34	0.154882	0.557587	0.303665	0.029*	
C73	0.7031 (4)	0.4146 (2)	0.6877 (2)	0.0294 (8)	
H73	0.763819	0.376018	0.706657	0.035*	
C45	0.8448 (4)	0.5850 (2)	1.16922 (19)	0.0263 (7)	
H45	0.876601	0.634614	1.162171	0.032*	
C44	0.8233 (4)	0.5609 (2)	1.24019 (19)	0.0299 (8)	
H44	0.836356	0.593981	1.281391	0.036*	
C64	0.4167 (4)	0.2389 (2)	0.7802 (2)	0.0344 (9)	
H64	0.365882	0.213393	0.740571	0.041*	
C51	1.0327 (5)	0.7779 (2)	0.9963 (2)	0.0383 (10)	
H51	1.074103	0.824520	0.985272	0.046*	
C52	0.8878 (5)	0.7695 (2)	0.9893 (2)	0.0379 (9)	
H52	0.829756	0.810759	0.974366	0.046*	
C30	0.1001 (5)	0.7451 (2)	0.2228 (2)	0.0383 (9)	
H30	0.090731	0.759946	0.172050	0.046*	
C26	0.4698 (4)	0.6771 (2)	0.39265 (19)	0.0271 (7)	
C15	0.1683 (4)	0.46846 (19)	0.41981 (19)	0.0243 (7)	
C56	1.0316 (4)	0.4508 (2)	0.9003 (2)	0.0376 (9)	
H56	0.998908	0.430854	0.944871	0.045*	
C37	0.4110 (4)	0.4983 (3)	0.1163 (2)	0.0430 (10)	
H37	0.499747	0.503265	0.096088	0.052*	
C18	0.0456 (4)	0.4993 (3)	0.5526 (2)	0.0372 (9)	
H18	0.004057	0.509763	0.597801	0.045*	
C80	0.6078 (4)	0.6682 (2)	0.8405 (2)	0.0384 (9)	
H80	0.688384	0.681893	0.815536	0.046*	
C50	1.1160 (5)	0.7185 (2)	1.0193 (2)	0.0374 (9)	
H50	1.214923	0.724742	1.025205	0.045*	
C66	0.5497 (4)	0.3476 (2)	0.8271 (2)	0.0264 (7)	
C65	0.4750 (4)	0.3076 (2)	0.7687 (2)	0.0303 (8)	
H65	0.465261	0.329003	0.720357	0.036*	
C49	1.0578 (4)	0.6495 (2)	1.0341 (2)	0.0308 (8)	
H49	1.116362	0.608789	1.049997	0.037*	
C16	0.2464 (4)	0.4628 (2)	0.4875 (2)	0.0340 (9)	
H16	0.342581	0.448950	0.488758	0.041*	
C27	0.3551 (4)	0.6373 (2)	0.35330 (19)	0.0258 (7)	
C79	0.5074 (5)	0.7219 (2)	0.8530 (3)	0.0420 (10)	
H79	0.520997	0.772144	0.837996	0.050*	
C20	0.0292 (4)	0.4909 (2)	0.4199 (2)	0.0349 (8)	
H20	−0.024785	0.496229	0.373790	0.042*	
C17	0.1843 (4)	0.4775 (3)	0.5538 (2)	0.0418 (10)	
H17	0.237876	0.472560	0.600113	0.050*	
C70	0.5280 (4)	0.5274 (2)	0.6315 (2)	0.0359 (9)	
H70	0.468396	0.566321	0.612105	0.043*	
C36	0.3967 (4)	0.5186 (2)	0.1900 (2)	0.0345 (9)	
H36	0.475133	0.537322	0.219705	0.041*	
C71	0.6249 (4)	0.4957 (2)	0.5871 (2)	0.0349 (8)	
H71	0.631979	0.512716	0.537486	0.042*	
C33	0.1266 (5)	0.7018 (2)	0.3705 (3)	0.0416 (10)	
H33	0.134528	0.687004	0.421334	0.050*	
C6	0.5339 (4)	0.3195 (2)	0.3607 (2)	0.0297 (8)	
C8	0.2790 (4)	0.3010 (2)	0.3128 (2)	0.0322 (9)	
C38	0.2981 (4)	0.4712 (2)	0.0719 (2)	0.0366 (9)	
H38	0.309431	0.456529	0.021909	0.044*	
C2	0.7682 (4)	0.3242 (2)	0.4240 (2)	0.0377 (9)	
H2	0.839901	0.351231	0.451724	0.045*	
C19	−0.0327 (4)	0.5057 (3)	0.4853 (2)	0.0375 (9)	
H19	−0.128561	0.520223	0.484044	0.045*	
C76	0.4718 (5)	0.5757 (2)	0.8972 (3)	0.0413 (10)	
H76	0.458972	0.525703	0.913038	0.050*	
C3	0.7889 (5)	0.2509 (2)	0.4087 (3)	0.0442 (11)	
H3	0.873844	0.227265	0.426483	0.053*	
C31	−0.0005 (4)	0.7652 (2)	0.2698 (3)	0.0439 (10)	
H31	−0.079287	0.793920	0.251571	0.053*	
C5	0.5607 (5)	0.2433 (2)	0.3439 (3)	0.0409 (10)	
H5	0.490525	0.215276	0.316146	0.049*	
C13	0.2118 (5)	0.3147 (2)	0.2426 (2)	0.0390 (9)	
H13	0.249212	0.351190	0.211223	0.047*	
C60	1.0152 (5)	0.5377 (3)	0.8013 (3)	0.0524 (13)	
H60	0.970105	0.578958	0.776480	0.063*	
C4	0.6850 (5)	0.2095 (2)	0.3666 (3)	0.0471 (11)	
H4	0.701263	0.158778	0.354298	0.057*	
C59	1.1261 (5)	0.5034 (3)	0.7699 (3)	0.0528 (12)	
H59	1.155975	0.520717	0.723702	0.063*	
C9	0.2289 (5)	0.2437 (2)	0.3556 (3)	0.0454 (11)	
H9	0.275373	0.231087	0.402356	0.054*	
C77	0.3692 (5)	0.6289 (3)	0.9078 (3)	0.0554 (13)	
H77	0.285425	0.614622	0.929463	0.066*	
C11	0.0388 (6)	0.2231 (3)	0.2615 (4)	0.0629 (15)	
H11	−0.046526	0.198648	0.245715	0.075*	
C10	0.1071 (6)	0.2044 (3)	0.3282 (4)	0.0640 (17)	
H10	0.072592	0.164545	0.356533	0.077*	
C12	0.0916 (6)	0.2760 (3)	0.2178 (3)	0.0585 (14)	
H12	0.046507	0.286672	0.170176	0.070*	
C32	0.0128 (5)	0.7439 (3)	0.3430 (3)	0.0493 (12)	
H32	−0.056947	0.758179	0.375289	0.059*	

### {2,2'-[(1S,2S)-1,2-Diphenylethane-1,2-diylbis(nitrilophenylmethanylylidene)]diphenolato}nickel(II) (1) Atomic displacement parameters (Å^2^)

**Table:**

	*U*^11^	*U*^22^	*U*^33^	*U*^12^	*U*^13^	*U*^23^
Ni1	0.0205 (2)	0.0260 (2)	0.0244 (2)	0.00201 (18)	0.00211 (16)	0.00040 (17)
Ni2	0.0223 (2)	0.0277 (2)	0.0219 (2)	−0.00443 (18)	0.00268 (16)	0.00311 (18)
O1	0.0281 (13)	0.0297 (12)	0.0289 (12)	0.0047 (11)	0.0011 (10)	−0.0016 (11)
O3	0.0303 (14)	0.0291 (12)	0.0260 (12)	−0.0032 (10)	0.0022 (10)	0.0051 (10)
O4	0.0340 (15)	0.0353 (14)	0.0281 (13)	−0.0107 (11)	0.0017 (11)	0.0050 (11)
N1	0.0254 (16)	0.0266 (17)	0.0240 (14)	0.0034 (12)	0.0063 (12)	0.0000 (11)
O2	0.0242 (14)	0.0315 (14)	0.0325 (14)	0.0012 (10)	0.0012 (11)	−0.0008 (11)
N4	0.0209 (15)	0.0268 (14)	0.0216 (14)	−0.0008 (11)	0.0031 (11)	0.0016 (11)
N3	0.0212 (15)	0.0285 (14)	0.0231 (15)	0.0005 (12)	0.0062 (12)	0.0031 (12)
N2	0.0209 (15)	0.0297 (16)	0.0223 (14)	−0.0008 (12)	0.0034 (12)	0.0001 (12)
C48	0.0242 (19)	0.0296 (18)	0.0191 (16)	−0.0012 (14)	0.0047 (13)	−0.0006 (13)
C21	0.030 (2)	0.0315 (19)	0.0227 (17)	−0.0023 (15)	0.0041 (15)	−0.0012 (14)
C28	0.024 (2)	0.0227 (17)	0.0310 (19)	−0.0012 (14)	0.0043 (15)	−0.0003 (14)
C67	0.0164 (16)	0.0337 (19)	0.0253 (17)	0.0022 (13)	0.0042 (13)	0.0004 (13)
C74	0.0222 (18)	0.0299 (18)	0.0217 (16)	−0.0031 (14)	0.0039 (13)	0.0018 (13)
C47	0.0146 (16)	0.0312 (17)	0.0232 (17)	0.0025 (13)	0.0025 (13)	0.0017 (14)
C43	0.0184 (17)	0.049 (2)	0.0237 (17)	0.0017 (16)	0.0031 (13)	0.0044 (16)
C14	0.0219 (18)	0.0269 (19)	0.0250 (16)	0.0008 (13)	0.0022 (13)	−0.0006 (13)
C7	0.029 (2)	0.0295 (18)	0.0238 (17)	0.0022 (15)	0.0106 (15)	0.0024 (14)
C29	0.027 (2)	0.0273 (18)	0.036 (2)	0.0008 (15)	0.0026 (16)	−0.0021 (15)
C68	0.0219 (17)	0.0310 (18)	0.0241 (16)	−0.0046 (14)	0.0020 (13)	−0.0034 (14)
C46	0.0132 (16)	0.0340 (18)	0.0240 (17)	0.0025 (13)	0.0053 (13)	0.0029 (14)
C63	0.025 (2)	0.039 (2)	0.047 (2)	−0.0084 (17)	0.0072 (18)	−0.0008 (17)
C57	0.038 (2)	0.038 (2)	0.043 (2)	0.0076 (17)	0.0055 (18)	0.0022 (17)
C58	0.027 (2)	0.052 (2)	0.045 (2)	0.0071 (19)	0.0119 (17)	0.007 (2)
C78	0.048 (3)	0.047 (2)	0.043 (2)	0.015 (2)	0.014 (2)	0.0007 (19)
C75	0.0258 (19)	0.0325 (18)	0.0223 (17)	0.0002 (14)	0.0028 (14)	0.0001 (14)
C54	0.0227 (18)	0.0270 (16)	0.0206 (16)	−0.0035 (13)	0.0049 (13)	0.0035 (13)
C41	0.0148 (16)	0.0360 (18)	0.0230 (17)	0.0022 (14)	0.0034 (13)	0.0025 (14)
C39	0.0261 (19)	0.0376 (18)	0.0281 (18)	0.0004 (15)	−0.0023 (15)	−0.0023 (15)
C22	0.027 (2)	0.039 (2)	0.039 (2)	−0.0051 (17)	−0.0009 (17)	−0.0011 (18)
C42	0.0221 (17)	0.0370 (18)	0.0284 (17)	−0.0015 (17)	0.0020 (14)	0.0050 (17)
C61	0.0169 (18)	0.0359 (19)	0.0330 (19)	−0.0034 (15)	0.0044 (15)	−0.0016 (15)
C69	0.0247 (18)	0.043 (2)	0.0277 (17)	0.0032 (17)	0.0018 (14)	−0.0036 (17)
C24	0.045 (3)	0.0311 (19)	0.035 (2)	−0.0026 (17)	0.0019 (18)	−0.0043 (16)
C35	0.0187 (17)	0.0260 (18)	0.0261 (16)	0.0036 (12)	0.0021 (13)	0.0029 (13)
C53	0.029 (2)	0.0310 (19)	0.0324 (19)	0.0024 (15)	0.0068 (16)	−0.0010 (15)
C62	0.027 (2)	0.039 (2)	0.040 (2)	−0.0097 (16)	0.0058 (17)	0.0043 (17)
C23	0.039 (2)	0.039 (2)	0.040 (2)	−0.0095 (18)	0.0013 (18)	−0.0057 (18)
C55	0.0206 (18)	0.033 (2)	0.0264 (17)	−0.0024 (14)	0.0050 (14)	0.0026 (14)
C25	0.034 (2)	0.0296 (18)	0.0309 (19)	0.0009 (16)	0.0017 (16)	−0.0004 (15)
C40	0.0219 (18)	0.034 (2)	0.0313 (18)	0.0025 (14)	0.0048 (14)	0.0017 (14)
C1	0.030 (2)	0.036 (2)	0.0260 (18)	0.0066 (16)	0.0104 (15)	0.0054 (15)
C72	0.036 (2)	0.0374 (18)	0.0303 (18)	−0.0074 (18)	0.0132 (15)	−0.0078 (17)
C34	0.0192 (17)	0.0276 (16)	0.0260 (17)	0.0012 (13)	0.0025 (13)	−0.0005 (13)
C73	0.0246 (19)	0.0304 (18)	0.0343 (19)	−0.0005 (14)	0.0091 (15)	−0.0030 (14)
C45	0.0193 (18)	0.0334 (18)	0.0263 (17)	0.0034 (14)	0.0014 (14)	0.0003 (14)
C44	0.0214 (18)	0.044 (2)	0.0244 (17)	0.0060 (15)	0.0023 (14)	−0.0008 (15)
C64	0.022 (2)	0.042 (2)	0.040 (2)	−0.0072 (16)	0.0091 (17)	−0.0098 (17)
C51	0.050 (3)	0.030 (2)	0.036 (2)	−0.0114 (18)	0.0140 (19)	−0.0041 (16)
C52	0.052 (3)	0.0266 (19)	0.036 (2)	0.0052 (18)	0.0055 (19)	−0.0023 (16)
C30	0.035 (2)	0.036 (2)	0.042 (2)	0.0030 (17)	−0.0075 (18)	0.0046 (17)
C26	0.030 (2)	0.0296 (18)	0.0220 (16)	−0.0015 (14)	0.0049 (14)	0.0023 (14)
C15	0.0213 (18)	0.0246 (15)	0.0277 (18)	−0.0004 (13)	0.0067 (14)	0.0014 (13)
C56	0.037 (2)	0.041 (2)	0.036 (2)	0.0047 (18)	0.0132 (17)	0.0083 (17)
C37	0.0223 (19)	0.073 (3)	0.035 (2)	0.002 (2)	0.0083 (16)	−0.007 (2)
C18	0.030 (2)	0.051 (2)	0.0323 (19)	−0.002 (2)	0.0132 (16)	−0.0087 (19)
C80	0.031 (2)	0.038 (2)	0.047 (2)	0.0046 (17)	0.0101 (18)	0.0147 (18)
C50	0.034 (2)	0.040 (2)	0.040 (2)	−0.0106 (18)	0.0140 (18)	−0.0076 (17)
C66	0.0173 (17)	0.0321 (18)	0.0306 (18)	0.0004 (14)	0.0076 (14)	−0.0026 (14)
C65	0.0216 (19)	0.040 (2)	0.0297 (18)	0.0000 (15)	0.0062 (15)	−0.0055 (15)
C49	0.0238 (19)	0.0348 (19)	0.0342 (19)	−0.0012 (15)	0.0052 (15)	−0.0047 (15)
C16	0.0210 (19)	0.051 (2)	0.0301 (19)	0.0073 (16)	0.0052 (15)	0.0043 (16)
C27	0.0256 (19)	0.0310 (19)	0.0215 (17)	−0.0012 (14)	0.0069 (14)	0.0006 (14)
C79	0.044 (3)	0.036 (2)	0.047 (2)	0.0090 (18)	0.008 (2)	0.0118 (18)
C20	0.0221 (19)	0.053 (2)	0.0293 (18)	−0.0022 (18)	0.0009 (14)	0.0032 (18)
C17	0.031 (2)	0.068 (3)	0.0259 (19)	0.0048 (19)	0.0016 (16)	0.0008 (18)
C70	0.032 (2)	0.044 (2)	0.030 (2)	0.0014 (17)	−0.0036 (16)	0.0028 (16)
C36	0.0217 (19)	0.051 (2)	0.0307 (19)	−0.0018 (15)	0.0027 (15)	−0.0008 (16)
C71	0.040 (2)	0.0411 (19)	0.0242 (17)	−0.0113 (19)	0.0044 (15)	−0.0008 (17)
C33	0.042 (3)	0.040 (2)	0.046 (2)	0.0091 (18)	0.019 (2)	0.0067 (18)
C6	0.030 (2)	0.0305 (18)	0.0293 (18)	0.0066 (15)	0.0106 (16)	0.0036 (14)
C8	0.034 (2)	0.0282 (19)	0.035 (2)	0.0039 (16)	0.0115 (17)	−0.0077 (16)
C38	0.031 (2)	0.052 (2)	0.0271 (19)	0.0092 (17)	0.0057 (16)	−0.0019 (16)
C2	0.027 (2)	0.044 (2)	0.043 (2)	0.0090 (17)	0.0089 (17)	0.0063 (18)
C19	0.0233 (19)	0.055 (2)	0.036 (2)	0.0060 (19)	0.0099 (15)	0.0016 (19)
C76	0.041 (2)	0.035 (2)	0.050 (2)	0.0012 (18)	0.025 (2)	0.0033 (18)
C3	0.032 (2)	0.044 (2)	0.058 (3)	0.0162 (19)	0.014 (2)	0.008 (2)
C31	0.024 (2)	0.038 (2)	0.069 (3)	0.0045 (17)	−0.001 (2)	0.005 (2)
C5	0.037 (2)	0.033 (2)	0.054 (3)	0.0053 (17)	0.012 (2)	−0.0010 (18)
C13	0.041 (2)	0.032 (2)	0.044 (2)	0.0060 (17)	−0.0001 (19)	−0.0127 (17)
C60	0.052 (3)	0.051 (3)	0.058 (3)	0.015 (2)	0.027 (2)	0.021 (2)
C4	0.041 (3)	0.029 (2)	0.073 (3)	0.0122 (18)	0.013 (2)	0.000 (2)
C59	0.047 (3)	0.065 (3)	0.049 (3)	0.015 (3)	0.022 (2)	0.015 (2)
C9	0.053 (3)	0.035 (2)	0.051 (2)	−0.0052 (19)	0.021 (2)	−0.0066 (18)
C77	0.050 (3)	0.052 (3)	0.068 (3)	0.005 (2)	0.036 (3)	−0.001 (2)
C11	0.046 (3)	0.056 (3)	0.087 (4)	−0.004 (2)	0.004 (3)	−0.030 (3)
C10	0.060 (4)	0.038 (3)	0.099 (5)	−0.015 (2)	0.044 (4)	−0.012 (3)
C12	0.049 (3)	0.054 (3)	0.071 (3)	0.010 (2)	−0.009 (3)	−0.033 (3)
C32	0.034 (3)	0.046 (2)	0.071 (3)	0.0133 (19)	0.023 (2)	0.006 (2)

### {2,2'-[(1S,2S)-1,2-Diphenylethane-1,2-diylbis(nitrilophenylmethanylylidene)]diphenolato}nickel(II) (1) Geometric parameters (Å, º)

**Table:**

Ni1—O1	1.833 (2)	C23—H23	0.9500
Ni1—O2	1.836 (3)	C55—C56	1.374 (5)
Ni1—N1	1.861 (3)	C55—C60	1.399 (5)
Ni1—N2	1.863 (3)	C25—H25	0.9500
Ni2—O3	1.847 (2)	C25—C26	1.419 (5)
Ni2—O4	1.825 (3)	C40—H40	0.9500
Ni2—N4	1.867 (3)	C1—C6	1.420 (6)
Ni2—N3	1.861 (3)	C1—C2	1.422 (6)
O1—C1	1.308 (5)	C72—H72	0.9500
O3—C41	1.312 (4)	C72—C73	1.378 (5)
O4—C61	1.303 (5)	C72—C71	1.375 (6)
N1—C14	1.478 (4)	C34—H34	1.0000
N1—C7	1.314 (5)	C73—H73	0.9500
O2—C21	1.311 (5)	C45—H45	0.9500
N4—C67	1.300 (5)	C45—C44	1.378 (5)
N4—C74	1.472 (4)	C44—H44	0.9500
N3—C47	1.297 (5)	C64—H64	0.9500
N3—C54	1.483 (4)	C64—C65	1.372 (5)
N2—C34	1.479 (5)	C51—H51	0.9500
N2—C27	1.303 (5)	C51—C52	1.389 (6)
C48—C47	1.495 (5)	C51—C50	1.374 (6)
C48—C53	1.392 (5)	C52—H52	0.9500
C48—C49	1.398 (5)	C30—H30	0.9500
C21—C22	1.416 (6)	C30—C31	1.373 (6)
C21—C26	1.420 (5)	C26—C27	1.448 (5)
C28—C29	1.383 (5)	C15—C16	1.386 (5)
C28—C27	1.502 (5)	C15—C20	1.388 (5)
C28—C33	1.397 (5)	C56—H56	0.9500
C67—C68	1.514 (5)	C37—H37	0.9500
C67—C66	1.440 (5)	C37—C36	1.391 (5)
C74—H74	1.0000	C37—C38	1.384 (6)
C74—C75	1.524 (5)	C18—H18	0.9500
C74—C54	1.540 (5)	C18—C17	1.379 (6)
C47—C46	1.453 (5)	C18—C19	1.382 (6)
C43—H43	0.9500	C80—H80	0.9500
C43—C42	1.370 (5)	C80—C79	1.388 (6)
C43—C44	1.403 (6)	C50—H50	0.9500
C14—H14	1.0000	C50—C49	1.387 (6)
C14—C34	1.537 (5)	C66—C65	1.421 (5)
C14—C15	1.530 (4)	C65—H65	0.9500
C7—C6	1.453 (5)	C49—H49	0.9500
C7—C8	1.495 (5)	C16—H16	0.9500
C29—H29	0.9500	C16—C17	1.396 (5)
C29—C30	1.394 (6)	C79—H79	0.9500
C68—C69	1.392 (5)	C20—H20	0.9500
C68—C73	1.399 (5)	C20—C19	1.380 (5)
C46—C41	1.423 (5)	C17—H17	0.9500
C46—C45	1.419 (5)	C70—H70	0.9500
C63—H63	0.9500	C70—C71	1.387 (6)
C63—C62	1.365 (6)	C36—H36	0.9500
C63—C64	1.400 (6)	C71—H71	0.9500
C57—H57	0.9500	C33—H33	0.9500
C57—C58	1.354 (6)	C33—C32	1.385 (6)
C57—C56	1.394 (6)	C6—C5	1.423 (5)
C58—H58	0.9500	C8—C13	1.397 (6)
C58—C59	1.373 (7)	C8—C9	1.389 (6)
C78—H78	0.9500	C38—H38	0.9500
C78—C79	1.382 (6)	C2—H2	0.9500
C78—C77	1.382 (7)	C2—C3	1.358 (6)
C75—C80	1.382 (5)	C19—H19	0.9500
C75—C76	1.387 (5)	C76—H76	0.9500
C54—H54	1.0000	C76—C77	1.390 (6)
C54—C55	1.517 (5)	C3—H3	0.9500
C41—C42	1.415 (5)	C3—C4	1.415 (7)
C39—H39	0.9500	C31—H31	0.9500
C39—C40	1.380 (5)	C31—C32	1.368 (7)
C39—C38	1.387 (5)	C5—H5	0.9500
C22—H22	0.9500	C5—C4	1.369 (6)
C22—C23	1.369 (6)	C13—H13	0.9500
C42—H42	0.9500	C13—C12	1.386 (7)
C61—C62	1.414 (5)	C60—H60	0.9500
C61—C66	1.426 (5)	C60—C59	1.379 (6)
C69—H69	0.9500	C4—H4	0.9500
C69—C70	1.388 (5)	C59—H59	0.9500
C24—H24	0.9500	C9—H9	0.9500
C24—C23	1.397 (6)	C9—C10	1.417 (8)
C24—C25	1.373 (6)	C77—H77	0.9500
C35—C40	1.389 (5)	C11—H11	0.9500
C35—C34	1.520 (5)	C11—C10	1.366 (9)
C35—C36	1.396 (5)	C11—C12	1.352 (9)
C53—H53	0.9500	C10—H10	0.9500
C53—C52	1.379 (6)	C12—H12	0.9500
C62—H62	0.9500	C32—H32	0.9500
			
O1—Ni1—N1	93.37 (13)	N2—C34—C35	113.3 (3)
O1—Ni1—O2	85.23 (11)	N2—C34—H34	109.5
O1—Ni1—N2	178.67 (12)	C14—C34—H34	109.5
O2—Ni1—N1	178.46 (13)	C35—C34—C14	110.7 (3)
O2—Ni1—N2	93.97 (12)	C35—C34—H34	109.5
N1—Ni1—N2	87.42 (12)	C68—C73—H73	120.1
O3—Ni2—N4	179.41 (13)	C72—C73—C68	119.9 (4)
O3—Ni2—N3	92.66 (12)	C72—C73—H73	120.1
O4—Ni2—O3	85.46 (11)	C46—C45—H45	119.1
O4—Ni2—N4	94.30 (12)	C44—C45—C46	121.8 (3)
O4—Ni2—N3	176.57 (13)	C44—C45—H45	119.1
N3—Ni2—N4	87.60 (13)	C43—C44—H44	120.6
C1—O1—Ni1	126.5 (2)	C45—C44—C43	118.8 (3)
C41—O3—Ni2	125.0 (2)	C45—C44—H44	120.6
C61—O4—Ni2	128.1 (2)	C63—C64—H64	120.6
C14—N1—Ni1	109.6 (2)	C65—C64—C63	118.8 (4)
C7—N1—Ni1	128.1 (3)	C65—C64—H64	120.6
C7—N1—C14	122.3 (3)	C52—C51—H51	120.2
C21—O2—Ni1	126.5 (2)	C50—C51—H51	120.2
C67—N4—Ni2	128.2 (2)	C50—C51—C52	119.7 (4)
C67—N4—C74	120.5 (3)	C53—C52—C51	120.1 (4)
C74—N4—Ni2	111.3 (2)	C53—C52—H52	119.9
C47—N3—Ni2	128.7 (2)	C51—C52—H52	119.9
C47—N3—C54	121.2 (3)	C29—C30—H30	119.8
C54—N3—Ni2	110.0 (2)	C31—C30—C29	120.3 (4)
C34—N2—Ni1	111.4 (2)	C31—C30—H30	119.8
C27—N2—Ni1	128.5 (3)	C21—C26—C27	122.1 (3)
C27—N2—C34	119.9 (3)	C25—C26—C21	118.6 (3)
C53—C48—C47	119.1 (3)	C25—C26—C27	119.2 (3)
C53—C48—C49	119.5 (3)	C16—C15—C14	124.1 (3)
C49—C48—C47	121.2 (3)	C16—C15—C20	118.4 (3)
O2—C21—C22	117.4 (4)	C20—C15—C14	117.5 (3)
O2—C21—C26	124.6 (3)	C57—C56—H56	119.3
C22—C21—C26	118.0 (3)	C55—C56—C57	121.4 (4)
C29—C28—C27	120.8 (3)	C55—C56—H56	119.3
C29—C28—C33	119.4 (4)	C36—C37—H37	119.4
C33—C28—C27	119.7 (3)	C38—C37—H37	119.4
N4—C67—C68	118.8 (3)	C38—C37—C36	121.1 (3)
N4—C67—C66	122.8 (3)	C17—C18—H18	120.1
C66—C67—C68	118.3 (3)	C17—C18—C19	119.8 (3)
N4—C74—H74	109.1	C19—C18—H18	120.1
N4—C74—C75	111.7 (3)	C75—C80—H80	119.4
N4—C74—C54	105.6 (3)	C75—C80—C79	121.3 (4)
C75—C74—H74	109.1	C79—C80—H80	119.4
C75—C74—C54	112.1 (3)	C51—C50—H50	119.5
C54—C74—H74	109.1	C51—C50—C49	121.1 (4)
N3—C47—C48	119.5 (3)	C49—C50—H50	119.5
N3—C47—C46	121.2 (3)	C61—C66—C67	121.5 (3)
C46—C47—C48	119.2 (3)	C65—C66—C67	120.0 (3)
C42—C43—H43	119.5	C65—C66—C61	118.4 (3)
C42—C43—C44	121.0 (3)	C64—C65—C66	121.9 (4)
C44—C43—H43	119.5	C64—C65—H65	119.0
N1—C14—H14	109.7	C66—C65—H65	119.0
N1—C14—C34	104.8 (3)	C48—C49—H49	120.4
N1—C14—C15	111.9 (3)	C50—C49—C48	119.2 (4)
C34—C14—H14	109.7	C50—C49—H49	120.4
C15—C14—H14	109.7	C15—C16—H16	119.9
C15—C14—C34	110.9 (3)	C15—C16—C17	120.2 (4)
N1—C7—C6	121.2 (3)	C17—C16—H16	119.9
N1—C7—C8	119.4 (3)	N2—C27—C28	120.0 (3)
C6—C7—C8	119.3 (3)	N2—C27—C26	121.5 (3)
C28—C29—H29	120.1	C26—C27—C28	118.5 (3)
C28—C29—C30	119.9 (4)	C78—C79—C80	120.1 (4)
C30—C29—H29	120.1	C78—C79—H79	120.0
C69—C68—C67	123.0 (3)	C80—C79—H79	120.0
C69—C68—C73	119.3 (3)	C15—C20—H20	119.2
C73—C68—C67	117.6 (3)	C19—C20—C15	121.7 (4)
C41—C46—C47	121.5 (3)	C19—C20—H20	119.2
C45—C46—C47	119.7 (3)	C18—C17—C16	120.4 (4)
C45—C46—C41	118.8 (3)	C18—C17—H17	119.8
C62—C63—H63	119.4	C16—C17—H17	119.8
C62—C63—C64	121.2 (4)	C69—C70—H70	119.7
C64—C63—H63	119.4	C71—C70—C69	120.6 (4)
C58—C57—H57	119.8	C71—C70—H70	119.7
C58—C57—C56	120.3 (4)	C35—C36—H36	120.2
C56—C57—H57	119.8	C37—C36—C35	119.7 (4)
C57—C58—H58	120.1	C37—C36—H36	120.2
C57—C58—C59	119.8 (4)	C72—C71—C70	119.3 (3)
C59—C58—H58	120.1	C72—C71—H71	120.3
C79—C78—H78	120.5	C70—C71—H71	120.3
C77—C78—H78	120.5	C28—C33—H33	120.2
C77—C78—C79	119.0 (4)	C32—C33—C28	119.6 (4)
C80—C75—C74	118.5 (3)	C32—C33—H33	120.2
C80—C75—C76	118.4 (4)	C1—C6—C7	121.5 (3)
C76—C75—C74	123.1 (3)	C1—C6—C5	118.6 (4)
N3—C54—C74	104.5 (3)	C5—C6—C7	119.8 (4)
N3—C54—H54	109.3	C13—C8—C7	119.7 (3)
N3—C54—C55	113.0 (3)	C9—C8—C7	121.7 (4)
C74—C54—H54	109.3	C9—C8—C13	118.5 (4)
C55—C54—C74	111.3 (3)	C39—C38—H38	120.5
C55—C54—H54	109.3	C37—C38—C39	119.1 (3)
O3—C41—C46	124.3 (3)	C37—C38—H38	120.5
O3—C41—C42	117.6 (3)	C1—C2—H2	119.2
C42—C41—C46	118.2 (3)	C3—C2—C1	121.6 (4)
C40—C39—H39	120.0	C3—C2—H2	119.2
C40—C39—C38	120.0 (4)	C18—C19—H19	120.2
C38—C39—H39	120.0	C20—C19—C18	119.5 (4)
C21—C22—H22	119.3	C20—C19—H19	120.2
C23—C22—C21	121.5 (4)	C75—C76—H76	119.8
C23—C22—H22	119.3	C75—C76—C77	120.4 (4)
C43—C42—C41	121.4 (4)	C77—C76—H76	119.8
C43—C42—H42	119.3	C2—C3—H3	119.7
C41—C42—H42	119.3	C2—C3—C4	120.6 (4)
O4—C61—C62	117.2 (3)	C4—C3—H3	119.7
O4—C61—C66	124.7 (3)	C30—C31—H31	120.0
C62—C61—C66	118.1 (3)	C32—C31—C30	119.9 (4)
C68—C69—H69	120.1	C32—C31—H31	120.0
C70—C69—C68	119.8 (3)	C6—C5—H5	119.2
C70—C69—H69	120.1	C4—C5—C6	121.7 (4)
C23—C24—H24	120.5	C4—C5—H5	119.2
C25—C24—H24	120.5	C8—C13—H13	119.3
C25—C24—C23	118.9 (4)	C12—C13—C8	121.4 (5)
C40—C35—C34	117.5 (3)	C12—C13—H13	119.3
C40—C35—C36	118.6 (3)	C55—C60—H60	119.3
C36—C35—C34	123.8 (3)	C59—C60—C55	121.5 (4)
C48—C53—H53	119.8	C59—C60—H60	119.3
C52—C53—C48	120.3 (4)	C3—C4—H4	120.4
C52—C53—H53	119.8	C5—C4—C3	119.2 (4)
C63—C62—C61	121.4 (4)	C5—C4—H4	120.4
C63—C62—H62	119.3	C58—C59—C60	119.9 (4)
C61—C62—H62	119.3	C58—C59—H59	120.1
C22—C23—C24	121.0 (4)	C60—C59—H59	120.1
C22—C23—H23	119.5	C8—C9—H9	120.7
C24—C23—H23	119.5	C8—C9—C10	118.7 (5)
C56—C55—C54	125.2 (3)	C10—C9—H9	120.7
C56—C55—C60	116.9 (3)	C78—C77—C76	120.8 (4)
C60—C55—C54	117.7 (3)	C78—C77—H77	119.6
C24—C25—H25	119.0	C76—C77—H77	119.6
C24—C25—C26	121.9 (4)	C10—C11—H11	119.7
C26—C25—H25	119.0	C12—C11—H11	119.7
C39—C40—C35	121.4 (3)	C12—C11—C10	120.6 (5)
C39—C40—H40	119.3	C9—C10—H10	119.6
C35—C40—H40	119.3	C11—C10—C9	120.9 (5)
O1—C1—C6	124.7 (3)	C11—C10—H10	119.6
O1—C1—C2	117.0 (4)	C13—C12—H12	120.1
C6—C1—C2	118.2 (3)	C11—C12—C13	119.8 (5)
C73—C72—H72	119.5	C11—C12—H12	120.1
C71—C72—H72	119.5	C33—C32—H32	119.6
C71—C72—C73	121.1 (3)	C31—C32—C33	120.9 (4)
N2—C34—C14	104.1 (3)	C31—C32—H32	119.6
			
Ni1—O1—C1—C6	−10.8 (5)	C7—C8—C9—C10	172.3 (4)
Ni1—O1—C1—C2	169.2 (2)	C29—C28—C27—N2	−79.9 (4)
Ni1—N1—C14—C34	42.8 (3)	C29—C28—C27—C26	97.9 (4)
Ni1—N1—C14—C15	−77.5 (3)	C29—C28—C33—C32	−0.3 (6)
Ni1—N1—C7—C6	9.8 (5)	C29—C30—C31—C32	0.0 (7)
Ni1—N1—C7—C8	−172.4 (2)	C68—C67—C66—C61	173.6 (3)
Ni1—O2—C21—C22	172.2 (3)	C68—C67—C66—C65	−9.1 (5)
Ni1—O2—C21—C26	−8.7 (5)	C68—C69—C70—C71	0.9 (6)
Ni1—N2—C34—C14	36.2 (3)	C46—C41—C42—C43	2.8 (5)
Ni1—N2—C34—C35	−84.1 (3)	C46—C45—C44—C43	2.5 (5)
Ni1—N2—C27—C28	−176.0 (2)	C63—C64—C65—C66	−1.0 (6)
Ni1—N2—C27—C26	6.3 (5)	C57—C58—C59—C60	5.2 (8)
Ni2—O3—C41—C46	−20.5 (5)	C58—C57—C56—C55	1.1 (7)
Ni2—O3—C41—C42	159.6 (2)	C75—C74—C54—N3	74.0 (3)
Ni2—O4—C61—C62	178.3 (3)	C75—C74—C54—C55	−163.8 (3)
Ni2—O4—C61—C66	−1.3 (5)	C75—C80—C79—C78	−2.0 (7)
Ni2—N4—C67—C68	−168.9 (2)	C75—C76—C77—C78	−1.9 (8)
Ni2—N4—C67—C66	8.2 (5)	C54—N3—C47—C48	4.2 (5)
Ni2—N4—C74—C75	−87.7 (3)	C54—N3—C47—C46	−176.2 (3)
Ni2—N4—C74—C54	34.4 (3)	C54—C74—C75—C80	71.4 (4)
Ni2—N3—C47—C48	−172.7 (2)	C54—C74—C75—C76	−110.8 (4)
Ni2—N3—C47—C46	6.9 (5)	C54—C55—C56—C57	178.3 (4)
Ni2—N3—C54—C74	41.6 (3)	C54—C55—C60—C59	−179.0 (5)
Ni2—N3—C54—C55	−79.5 (3)	C41—C46—C45—C44	−2.1 (5)
O1—Ni1—N1—C14	160.0 (2)	C22—C21—C26—C25	−4.2 (5)
O1—Ni1—N1—C7	−20.8 (3)	C22—C21—C26—C27	172.2 (3)
O1—Ni1—O2—C21	−162.1 (3)	C42—C43—C44—C45	−0.2 (5)
O1—C1—C6—C7	−7.7 (5)	C61—C66—C65—C64	−1.8 (5)
O1—C1—C6—C5	176.0 (3)	C69—C68—C73—C72	0.3 (5)
O1—C1—C2—C3	−177.6 (4)	C69—C70—C71—C72	0.1 (6)
O3—Ni2—O4—C61	−176.6 (3)	C24—C25—C26—C21	3.2 (5)
O3—Ni2—N3—C47	−21.6 (3)	C24—C25—C26—C27	−173.3 (3)
O3—Ni2—N3—C54	161.2 (2)	C53—C48—C47—C46	−101.7 (4)
O3—C41—C42—C43	−177.3 (3)	C53—C48—C49—C50	−1.9 (5)
O4—Ni2—O3—C41	−155.2 (3)	C62—C63—C64—C65	2.4 (6)
O4—Ni2—N4—C67	−7.6 (3)	C62—C61—C66—C67	−179.6 (3)
O4—Ni2—N4—C74	173.3 (2)	C62—C61—C66—C65	3.1 (5)
O4—C61—C62—C63	178.7 (4)	C23—C24—C25—C26	0.0 (6)
O4—C61—C66—C67	−0.1 (5)	C55—C60—C59—C58	−0.6 (9)
O4—C61—C66—C65	−177.4 (3)	C25—C24—C23—C22	−2.2 (6)
N1—Ni1—O1—C1	20.9 (3)	C25—C26—C27—N2	−175.6 (3)
N1—Ni1—N2—C34	−11.0 (2)	C25—C26—C27—C28	6.7 (5)
N1—Ni1—N2—C27	163.4 (3)	C40—C39—C38—C37	−1.3 (6)
N1—C14—C34—N2	−49.6 (3)	C40—C35—C34—N2	−170.5 (3)
N1—C14—C34—C35	72.5 (3)	C40—C35—C34—C14	72.9 (4)
N1—C14—C15—C16	8.3 (5)	C40—C35—C36—C37	−1.7 (6)
N1—C14—C15—C20	−173.6 (3)	C1—C6—C5—C4	2.5 (6)
N1—C7—C6—C1	8.1 (5)	C1—C2—C3—C4	1.0 (6)
N1—C7—C6—C5	−175.7 (3)	C34—N2—C27—C28	−2.0 (4)
N1—C7—C8—C13	61.6 (5)	C34—N2—C27—C26	−179.7 (3)
N1—C7—C8—C9	−114.3 (4)	C34—C14—C15—C16	−108.3 (4)
N2—Ni1—N1—C14	−19.0 (2)	C34—C14—C15—C20	69.7 (4)
N2—Ni1—N1—C7	160.3 (3)	C34—C35—C40—C39	−176.1 (3)
N2—Ni1—O2—C21	16.8 (3)	C34—C35—C36—C37	176.0 (4)
N2—C27—C28—C33	101.6 (4)	C73—C68—C69—C70	−1.1 (6)
O2—Ni1—O1—C1	−159.8 (3)	C73—C72—C71—C70	−0.9 (6)
O2—Ni1—N2—C34	169.7 (2)	C45—C46—C41—O3	179.5 (3)
O2—Ni1—N2—C27	−15.9 (3)	C45—C46—C41—C42	−0.5 (5)
O2—C21—C22—C23	−178.7 (4)	C44—C43—C42—C41	−2.4 (5)
O2—C21—C26—C25	176.7 (3)	C64—C63—C62—C61	−1.0 (6)
O2—C21—C26—C27	−6.9 (5)	C51—C50—C49—C48	0.0 (6)
N3—Ni2—O3—C41	27.7 (3)	C52—C51—C50—C49	1.5 (6)
N3—Ni2—N4—C67	169.6 (3)	C30—C31—C32—C33	−0.3 (7)
N3—Ni2—N4—C74	−9.6 (2)	C26—C21—C22—C23	2.2 (6)
N3—C47—C46—C41	10.0 (5)	C15—C14—C34—N2	71.3 (3)
N3—C47—C46—C45	−172.4 (3)	C15—C14—C34—C35	−166.6 (3)
N3—C54—C74—N4	−47.8 (3)	C15—C16—C17—C18	−1.4 (7)
N3—C54—C55—C56	9.9 (5)	C15—C20—C19—C18	1.0 (7)
N3—C54—C55—C60	−175.0 (4)	C56—C57—C58—C59	−5.4 (7)
N3—C47—C48—C53	77.9 (4)	C56—C55—C60—C59	−3.5 (7)
N4—Ni2—O4—C61	4.0 (3)	C80—C75—C76—C77	−0.7 (7)
N4—Ni2—N3—C47	157.9 (3)	C50—C51—C52—C53	−1.2 (6)
N4—Ni2—N3—C54	−19.3 (2)	C66—C67—C68—C69	108.1 (4)
N4—C67—C68—C69	−74.6 (5)	C66—C67—C68—C73	−74.8 (4)
N4—C67—C68—C73	102.4 (4)	C66—C61—C62—C63	−1.8 (6)
N4—C67—C66—C61	−3.5 (5)	C49—C48—C47—N3	−97.4 (4)
N4—C67—C66—C65	173.7 (3)	C49—C48—C47—C46	83.0 (4)
N4—C74—C75—C80	−170.3 (3)	C49—C48—C53—C52	2.2 (5)
N4—C74—C75—C76	7.5 (5)	C16—C15—C20—C19	−1.6 (6)
N4—C74—C54—C55	74.4 (3)	C27—N2—C34—C14	−138.7 (3)
C48—C47—C46—C41	−170.4 (3)	C27—N2—C34—C35	100.9 (3)
C48—C47—C46—C45	7.2 (5)	C27—C28—C29—C30	−178.5 (4)
C48—C53—C52—C51	−0.6 (6)	C27—C28—C33—C32	178.3 (4)
C21—C22—C23—C24	1.1 (6)	C79—C78—C77—C76	2.6 (8)
C21—C26—C27—N2	8.1 (5)	C20—C15—C16—C17	1.8 (6)
C21—C26—C27—C28	−169.7 (3)	C17—C18—C19—C20	−0.5 (7)
C28—C29—C30—C31	0.1 (6)	C36—C35—C40—C39	1.7 (5)
C28—C33—C32—C31	0.4 (7)	C36—C35—C34—N2	11.8 (5)
C67—N4—C74—C75	93.1 (4)	C36—C35—C34—C14	−104.8 (4)
C67—N4—C74—C54	−144.8 (3)	C36—C37—C38—C39	1.3 (7)
C67—C68—C69—C70	175.9 (3)	C71—C72—C73—C68	0.7 (6)
C67—C68—C73—C72	−176.8 (3)	C33—C28—C29—C30	0.0 (6)
C67—C66—C65—C64	−179.1 (3)	C33—C28—C27—C26	−80.7 (4)
C74—N4—C67—C68	10.2 (5)	C6—C7—C8—C13	−120.6 (4)
C74—N4—C67—C66	−172.7 (3)	C6—C7—C8—C9	63.5 (5)
C74—C75—C80—C79	−179.4 (4)	C6—C1—C2—C3	2.4 (6)
C74—C75—C76—C77	−178.6 (4)	C6—C5—C4—C3	0.8 (7)
C74—C54—C55—C56	−107.2 (4)	C8—C7—C6—C1	−169.7 (3)
C74—C54—C55—C60	67.8 (5)	C8—C7—C6—C5	6.6 (5)
C47—N3—C54—C74	−135.8 (3)	C8—C13—C12—C11	−1.1 (7)
C47—N3—C54—C55	103.1 (4)	C8—C9—C10—C11	−1.0 (7)
C47—C48—C53—C52	−173.2 (3)	C38—C39—C40—C35	−0.2 (6)
C47—C48—C49—C50	173.4 (3)	C38—C37—C36—C35	0.2 (7)
C47—C46—C41—O3	−2.8 (5)	C2—C1—C6—C7	172.3 (3)
C47—C46—C41—C42	177.1 (3)	C2—C1—C6—C5	−4.0 (5)
C47—C46—C45—C44	−179.8 (3)	C2—C3—C4—C5	−2.6 (7)
C14—N1—C7—C6	−171.0 (3)	C19—C18—C17—C16	0.7 (7)
C14—N1—C7—C8	6.8 (5)	C76—C75—C80—C79	2.7 (7)
C14—C15—C16—C17	179.8 (4)	C13—C8—C9—C10	−3.6 (6)
C14—C15—C20—C19	−179.8 (4)	C60—C55—C56—C57	3.3 (6)
C7—N1—C14—C34	−136.6 (3)	C9—C8—C13—C12	4.7 (6)
C7—N1—C14—C15	103.2 (3)	C77—C78—C79—C80	−0.7 (7)
C7—C6—C5—C4	−173.9 (4)	C10—C11—C12—C13	−3.7 (7)
C7—C8—C13—C12	−171.3 (4)	C12—C11—C10—C9	4.7 (8)

### {2,2'-[(1S,2S)-1,2-Diphenylethane-1,2-diylbis(nitrilophenylmethanylylidene)]diphenolato}nickel(II) (1) Hydrogen-bond geometry (Å, º)

**Table:**

*D*—H···*A*	*D*—H	H···*A*	*D*···*A*	*D*—H···*A*
C43—H43···O1^i^	0.95	2.60	3.500 (4)	159
C51—H51···O3^ii^	0.95	2.52	3.417 (5)	158
C71—H71···O1	0.95	2.63	3.308 (4)	128
C71—H71···O2	0.95	2.62	3.562 (4)	173

Symmetry codes: (i) *x*, *y*, *z*+1; (ii) −*x*+2, *y*+1/2, −*z*+2.

### {2,2'-[(1S,2S)-1,2-Diphenylethane-1,2-diylbis(nitrilophenylmethanylylidene)]diphenolato}nickel(II) ethanol disolvate (1_EtOH) Crystal data

**Table:**

[Ni(C_40_H_30_N_2_O_2_)]·2C_2_H_6_O	*Z* = 2
*M**_r_* = 721.50	*F*(000) = 760
Triclinic, *P*1	*D*_x_ = 1.331 Mg m^−^^3^
*a* = 10.5830 (2) Å	Mo *K*α radiation, λ = 0.71073 Å
*b* = 12.4110 (2) Å	Cell parameters from 24617 reflections
*c* = 13.9837 (2) Å	θ = 3.0–31.3°
α = 94.352 (1)°	µ = 0.59 mm^−^^1^
β = 100.599 (1)°	*T* = 120 K
γ = 90.584 (1)°	Platelet, brown
*V* = 1799.61 (5) Å^3^	0.26 × 0.20 × 0.09 mm

### {2,2'-[(1S,2S)-1,2-Diphenylethane-1,2-diylbis(nitrilophenylmethanylylidene)]diphenolato}nickel(II) ethanol disolvate (1_EtOH) Data collection

**Table:**

Rigaku Oxford Diffraction, Synergy Custom system, HyPix diffractometer	13032 independent reflections
Radiation source: fine-focus sealed X-ray tube, Enhance (Mo) X-ray Source	12354 reflections with *I* > 2σ(*I*)
Graphite monochromator	*R*_int_ = 0.025
ω scans	θ_max_ = 25.4°, θ_min_ = 2.7°
Absorption correction: multi-scan (CrysAlisPro; Rigaku OD, 2018)	*h* = −12→12
*T*_min_ = 0.824, *T*_max_ = 1.000	*k* = −14→14
24831 measured reflections	*l* = −16→16

### {2,2'-[(1S,2S)-1,2-Diphenylethane-1,2-diylbis(nitrilophenylmethanylylidene)]diphenolato}nickel(II) ethanol disolvate (1_EtOH) Refinement

**Table:**

Refinement on *F*^2^	Hydrogen site location: inferred from neighbouring sites
Least-squares matrix: full	H-atom parameters constrained
*R*[*F*^2^ > 2σ(*F*^2^)] = 0.035	*w* = 1/[σ^2^(*F*_o_^2^) + (0.042*P*)^2^ + 0.5385*P*] where *P* = (*F*_o_^2^ + 2*F*_c_^2^)/3
*wR*(*F*^2^) = 0.083	(Δ/σ)_max_ < 0.001
*S* = 1.03	Δρ_max_ = 0.32 e Å^−^^3^
13032 reflections	Δρ_min_ = −0.37 e Å^−^^3^
938 parameters	Absolute structure: Flack *x* determined using 5653 quotients [(*I*^+^)-(*I*^-^)]/[(*I*^+^)+(*I*^-^)] (Parsons *et al.*, 2013)
9 restraints	Absolute structure parameter: −0.012 (5)
Primary atom site location: dual	

### {2,2'-[(1S,2S)-1,2-Diphenylethane-1,2-diylbis(nitrilophenylmethanylylidene)]diphenolato}nickel(II) ethanol disolvate (1_EtOH) Special details

**Table:**

Geometry. All esds (except the esd in the dihedral angle between two l.s. planes) are estimated using the full covariance matrix. The cell esds are taken into account individually in the estimation of esds in distances, angles and torsion angles; correlations between esds in cell parameters are only used when they are defined by crystal symmetry. An approximate (isotropic) treatment of cell esds is used for estimating esds involving l.s. planes.

### {2,2'-[(1S,2S)-1,2-Diphenylethane-1,2-diylbis(nitrilophenylmethanylylidene)]diphenolato}nickel(II) ethanol disolvate (1_EtOH) Fractional atomic coordinates and isotropic or equivalent isotropic displacement parameters (Å^2^)

**Table:**

	*x*	*y*	*z*	*U*_iso_*/*U*_eq_	Occ. (<1)
Ni1	0.37805 (4)	0.27140 (3)	0.25629 (3)	0.02409 (12)	
O1	0.4813 (2)	0.3855 (2)	0.2414 (2)	0.0317 (7)	
O2	0.5302 (3)	0.2102 (2)	0.3071 (2)	0.0314 (7)	
N1	0.2258 (3)	0.3374 (3)	0.2050 (2)	0.0221 (7)	
N2	0.2778 (3)	0.1561 (3)	0.2786 (2)	0.0233 (7)	
C1	0.4456 (4)	0.4831 (3)	0.2189 (3)	0.0215 (8)	
C2	0.5439 (4)	0.5594 (3)	0.2161 (3)	0.0270 (8)	
H2	0.631150	0.538864	0.230451	0.032*	
C3	0.5155 (4)	0.6636 (3)	0.1929 (3)	0.0264 (8)	
H3	0.583229	0.713299	0.189791	0.032*	
C4	0.3891 (4)	0.6964 (3)	0.1740 (3)	0.0272 (8)	
H4	0.370460	0.768725	0.159354	0.033*	
C5	0.2915 (4)	0.6243 (3)	0.1766 (3)	0.0254 (8)	
H5	0.205193	0.647562	0.164190	0.030*	
C6	0.3159 (4)	0.5156 (3)	0.1975 (3)	0.0212 (8)	
C7	0.2090 (3)	0.4386 (3)	0.1900 (3)	0.0210 (8)	
C8	0.0764 (3)	0.4821 (3)	0.1651 (3)	0.0203 (7)	
C9	0.0225 (4)	0.4998 (3)	0.0697 (3)	0.0291 (8)	
H9	0.065528	0.477353	0.017890	0.035*	
C10	−0.0945 (4)	0.5506 (4)	0.0502 (3)	0.0342 (9)	
H10	−0.131263	0.563576	−0.015030	0.041*	
C11	−0.1579 (4)	0.5823 (3)	0.1251 (3)	0.0313 (10)	
H11	−0.237496	0.617825	0.111324	0.038*	
C12	−0.1060 (4)	0.5625 (3)	0.2202 (3)	0.0285 (9)	
H12	−0.150819	0.582719	0.271457	0.034*	
C13	0.0116 (3)	0.5130 (3)	0.2404 (3)	0.0253 (8)	
H13	0.048085	0.500214	0.305719	0.030*	
C14	0.1147 (3)	0.2607 (3)	0.1879 (3)	0.0218 (7)	
H14	0.034710	0.301503	0.192207	0.026*	
C15	0.0976 (3)	0.1957 (3)	0.0896 (3)	0.0241 (8)	
C16	0.1895 (4)	0.1919 (3)	0.0303 (3)	0.0331 (9)	
H16	0.267752	0.232459	0.050153	0.040*	
C17	0.1673 (5)	0.1291 (4)	−0.0577 (3)	0.0420 (11)	
H17	0.230819	0.126927	−0.097734	0.050*	
C18	0.0549 (5)	0.0699 (4)	−0.0877 (3)	0.0428 (11)	
H18	0.040694	0.026999	−0.148092	0.051*	
C19	−0.0372 (4)	0.0733 (4)	−0.0299 (3)	0.0392 (10)	
H19	−0.115281	0.032565	−0.050327	0.047*	
C20	−0.0162 (4)	0.1362 (3)	0.0587 (3)	0.0319 (9)	
H20	−0.080260	0.138343	0.098267	0.038*	
C21	0.5482 (4)	0.1092 (3)	0.3277 (3)	0.0251 (8)	
C22	0.6764 (4)	0.0788 (3)	0.3597 (3)	0.0301 (9)	
H22	0.743880	0.131644	0.367930	0.036*	
C23	0.7048 (4)	−0.0261 (3)	0.3790 (3)	0.0318 (9)	
H23	0.791833	−0.044842	0.399777	0.038*	
C24	0.6079 (4)	−0.1051 (3)	0.3685 (3)	0.0296 (9)	
H24	0.628426	−0.177823	0.380396	0.036*	
C25	0.4811 (4)	−0.0761 (3)	0.3405 (3)	0.0259 (8)	
H25	0.414633	−0.129621	0.334423	0.031*	
C26	0.4483 (4)	0.0312 (3)	0.3209 (3)	0.0228 (8)	
C27	0.3136 (4)	0.0583 (3)	0.2996 (3)	0.0237 (9)	
C28	0.2135 (4)	−0.0254 (3)	0.3047 (3)	0.0236 (8)	
C29	0.1823 (5)	−0.0492 (4)	0.3935 (3)	0.0364 (10)	
H29	0.228423	−0.014493	0.452735	0.044*	
C30	0.0849 (5)	−0.1229 (4)	0.3958 (4)	0.0451 (11)	
H30	0.063859	−0.138161	0.456666	0.054*	
C31	0.0176 (4)	−0.1748 (3)	0.3107 (3)	0.0385 (10)	
H31	−0.050883	−0.224176	0.312653	0.046*	
C32	0.0504 (4)	−0.1546 (3)	0.2235 (3)	0.0359 (10)	
H32	0.006985	−0.192766	0.165062	0.043*	
C33	0.1469 (4)	−0.0786 (3)	0.2194 (3)	0.0311 (9)	
H33	0.166833	−0.063435	0.158240	0.043 (13)*	
C34	0.1424 (3)	0.1882 (3)	0.2728 (3)	0.0238 (8)	
H34	0.084508	0.122567	0.258456	0.029*	
C35	0.1209 (4)	0.2499 (3)	0.3661 (3)	0.0264 (8)	
C36	0.2198 (4)	0.2979 (3)	0.4362 (3)	0.0333 (9)	
H36	0.306767	0.287389	0.429923	0.040*	
C37	0.1917 (5)	0.3609 (4)	0.5147 (3)	0.0458 (12)	
H37	0.259905	0.393668	0.562048	0.055*	
C38	0.0672 (5)	0.3770 (5)	0.5259 (3)	0.0483 (12)	
H38	0.049334	0.419475	0.581071	0.058*	
C39	−0.0320 (4)	0.3310 (4)	0.4564 (3)	0.0412 (11)	
H39	−0.118763	0.342851	0.462726	0.049*	
C40	−0.0048 (4)	0.2682 (4)	0.3783 (3)	0.0335 (9)	
H40	−0.073736	0.236287	0.331046	0.040*	
Ni2	0.76182 (4)	0.73251 (4)	0.75057 (4)	0.03064 (13)	
O3	0.8974 (3)	0.8003 (3)	0.7123 (3)	0.0482 (9)	
O4	0.8745 (3)	0.6230 (3)	0.7739 (3)	0.0488 (9)	
N3	0.6493 (3)	0.8439 (3)	0.7226 (2)	0.0240 (7)	
N4	0.6308 (3)	0.6619 (3)	0.7954 (2)	0.0238 (7)	
C41	0.9027 (4)	0.8977 (3)	0.6847 (3)	0.0299 (9)	
C42	1.0197 (4)	0.9319 (3)	0.6587 (3)	0.0342 (9)	
H42	1.088542	0.883083	0.660401	0.041*	
C43	1.0345 (4)	1.0333 (4)	0.6314 (3)	0.0361 (10)	
H43	1.113548	1.054349	0.614516	0.043*	
C44	0.9363 (4)	1.1065 (4)	0.6279 (3)	0.0376 (10)	
H44	0.948139	1.177284	0.609017	0.045*	
C45	0.8213 (4)	1.0760 (3)	0.6520 (3)	0.0309 (9)	
H45	0.754196	1.126574	0.649777	0.037*	
C46	0.8010 (4)	0.9712 (3)	0.6798 (3)	0.0265 (9)	
C47	0.6754 (4)	0.9404 (3)	0.6985 (3)	0.0235 (8)	
C48	0.5721 (4)	1.0231 (3)	0.6894 (3)	0.0253 (8)	
C49	0.5034 (4)	1.0435 (3)	0.5978 (3)	0.0309 (9)	
H49	0.522221	1.006171	0.540404	0.037*	
C50	0.4073 (4)	1.1189 (3)	0.5917 (4)	0.0433 (12)	
H50	0.360494	1.133869	0.529553	0.052*	
C51	0.3789 (4)	1.1725 (3)	0.6746 (4)	0.0429 (11)	
H51	0.311755	1.223156	0.669524	0.051*	
C52	0.4476 (4)	1.1526 (3)	0.7649 (3)	0.0376 (10)	
H52	0.428117	1.190581	0.821800	0.045*	
C53	0.5448 (4)	1.0779 (3)	0.7738 (3)	0.0292 (8)	
H53	0.592042	1.064370	0.836146	0.035*	
C54	0.5159 (3)	0.8106 (3)	0.7261 (3)	0.0223 (7)	
H54	0.466708	0.875924	0.741805	0.027*	
C55	0.4477 (4)	0.7533 (3)	0.6300 (3)	0.0254 (8)	
C56	0.5045 (4)	0.7357 (3)	0.5492 (3)	0.0325 (9)	
H56	0.590245	0.760853	0.551713	0.039*	
C57	0.4373 (5)	0.6815 (4)	0.4638 (3)	0.0415 (11)	
H57	0.477314	0.669750	0.408551	0.050*	
C58	0.3139 (5)	0.6451 (4)	0.4593 (3)	0.0483 (12)	
H58	0.267934	0.607989	0.401157	0.058*	
C59	0.2565 (5)	0.6628 (5)	0.5400 (4)	0.0516 (13)	
H59	0.170973	0.636969	0.537180	0.062*	
C60	0.3213 (4)	0.7171 (4)	0.6241 (3)	0.0375 (10)	
H60	0.279805	0.730052	0.678447	0.045*	
C61	0.8470 (4)	0.5231 (3)	0.7877 (3)	0.0304 (9)	
C62	0.9459 (4)	0.4474 (3)	0.7869 (3)	0.0341 (9)	
H62	1.026662	0.470310	0.774143	0.041*	
C63	0.9276 (4)	0.3417 (3)	0.8041 (3)	0.0337 (9)	
H63	0.995831	0.292614	0.803543	0.040*	
C64	0.8092 (4)	0.3052 (3)	0.8226 (3)	0.0352 (10)	
H64	0.796954	0.232151	0.835520	0.042*	
C65	0.7116 (4)	0.3769 (3)	0.8216 (3)	0.0300 (9)	
H65	0.630713	0.351692	0.832403	0.036*	
C66	0.7262 (4)	0.4867 (3)	0.8052 (3)	0.0233 (8)	
C67	0.6232 (3)	0.5606 (3)	0.8118 (3)	0.0218 (8)	
C68	0.5042 (3)	0.5155 (3)	0.8391 (3)	0.0212 (7)	
C69	0.4970 (4)	0.5023 (3)	0.9357 (3)	0.0307 (9)	
H69	0.565764	0.527673	0.986355	0.037*	
C70	0.3899 (4)	0.4524 (4)	0.9583 (3)	0.0361 (10)	
H70	0.385847	0.443278	1.024554	0.043*	
C71	0.2892 (4)	0.4157 (3)	0.8856 (3)	0.0293 (9)	
H71	0.216361	0.380685	0.901379	0.035*	
C72	0.2950 (4)	0.4304 (3)	0.7896 (3)	0.0284 (9)	
H72	0.225185	0.406369	0.739259	0.034*	
C73	0.4012 (4)	0.4795 (3)	0.7662 (3)	0.0268 (8)	
H73	0.404223	0.488871	0.699874	0.032*	
C74	0.5280 (3)	0.7367 (3)	0.8108 (3)	0.0223 (7)	
H74	0.445604	0.694473	0.805312	0.027*	
C75	0.5567 (4)	0.8020 (3)	0.9096 (3)	0.0256 (8)	
C76	0.6752 (4)	0.8066 (3)	0.9710 (3)	0.0333 (9)	
H76	0.743909	0.766005	0.952999	0.040*	
C77	0.6946 (4)	0.8702 (4)	1.0589 (3)	0.0407 (10)	
H77	0.776678	0.872822	1.100339	0.049*	
C78	0.5963 (5)	0.9292 (4)	1.0863 (3)	0.0404 (11)	
H78	0.610292	0.972316	1.146523	0.048*	
C79	0.4772 (4)	0.9257 (3)	1.0260 (3)	0.0378 (10)	
H79	0.408963	0.966589	1.044592	0.045*	
C80	0.4572 (4)	0.8626 (3)	0.9382 (3)	0.0310 (9)	
H80	0.375001	0.860397	0.896971	0.037*	
O5	1.1514 (3)	0.7195 (4)	0.8009 (2)	0.0552 (10)	
H5A	1.073735	0.722717	0.774226	0.083*	
C81	1.1594 (4)	0.6932 (4)	0.8981 (3)	0.0397 (10)	
H81A	1.250577	0.682702	0.927315	0.048*	
H81B	1.111845	0.624147	0.898573	0.048*	
C82	1.1053 (5)	0.7794 (4)	0.9591 (4)	0.0518 (13)	
H82A	1.014279	0.788522	0.931638	0.078*	
H82B	1.152657	0.847727	0.959489	0.078*	
H82C	1.113520	0.758244	1.026066	0.078*	
O6	0.6563 (3)	0.4437 (3)	0.4264 (3)	0.0631 (10)	
H6	0.616468	0.410607	0.374902	0.095*	
C83	0.5878 (6)	0.4284 (5)	0.5061 (5)	0.0665 (16)	
H83A	0.603680	0.492021	0.554258	0.080*	
H83B	0.494265	0.422062	0.480240	0.080*	
C84	0.6300 (7)	0.3319 (5)	0.5538 (4)	0.0633 (15)	
H84A	0.615917	0.269109	0.505871	0.095*	
H84B	0.580757	0.321610	0.605480	0.095*	
H84C	0.721670	0.339712	0.582326	0.095*	
O8	0.7474 (5)	0.6486 (4)	0.4071 (3)	0.0781 (13)	
H8	0.721227	0.584799	0.409037	0.117*	
C87	0.8313 (9)	0.6751 (7)	0.4834 (5)	0.094 (2)	
H87A	0.781335	0.697657	0.534514	0.113*	0.64 (4)
H87B	0.874004	0.741719	0.468923	0.113*	0.64 (4)
H87C	0.804246	0.744197	0.512747	0.113*	0.36 (4)
H87D	0.912800	0.690684	0.461343	0.113*	0.36 (4)
C88	0.9232 (19)	0.6179 (17)	0.526 (2)	0.136 (11)	0.64 (4)
H88A	0.986899	0.605775	0.484041	0.204*	0.64 (4)
H88B	0.887977	0.548252	0.539026	0.204*	0.64 (4)
H88C	0.964319	0.656465	0.588068	0.204*	0.64 (4)
C89	0.862 (3)	0.6013 (15)	0.5640 (17)	0.074 (9)	0.36 (4)
H89A	0.797807	0.608573	0.606047	0.110*	0.36 (4)
H89B	0.947560	0.620675	0.602587	0.110*	0.36 (4)
H89C	0.861724	0.526443	0.536042	0.110*	0.36 (4)
O7	0.7395 (3)	0.2614 (3)	0.1992 (2)	0.0543 (9)	
H7	0.675490	0.253105	0.225864	0.082*	
C85	0.6989 (4)	0.3014 (4)	0.1065 (3)	0.0400 (10)	
H85A	0.775200	0.316644	0.077524	0.048*	
H85B	0.653937	0.370125	0.114508	0.048*	
C86	0.6110 (6)	0.2224 (4)	0.0389 (4)	0.0546 (13)	
H86A	0.584629	0.252714	−0.024268	0.065*	
H86B	0.534772	0.208026	0.066924	0.065*	
H86C	0.655914	0.154869	0.029670	0.065*	

### {2,2'-[(1S,2S)-1,2-Diphenylethane-1,2-diylbis(nitrilophenylmethanylylidene)]diphenolato}nickel(II) ethanol disolvate (1_EtOH) Atomic displacement parameters (Å^2^)

**Table:**

	*U*^11^	*U*^22^	*U*^33^	*U*^12^	*U*^13^	*U*^23^
Ni1	0.0180 (2)	0.0213 (2)	0.0335 (3)	−0.00020 (18)	0.00330 (19)	0.00946 (19)
O1	0.0201 (14)	0.0265 (16)	0.0500 (18)	0.0006 (11)	0.0053 (13)	0.0157 (13)
O2	0.0203 (14)	0.0252 (15)	0.0493 (18)	−0.0008 (11)	0.0025 (13)	0.0162 (13)
N1	0.0193 (16)	0.0210 (17)	0.0270 (17)	0.0006 (13)	0.0052 (13)	0.0062 (13)
N2	0.0191 (16)	0.0221 (18)	0.0285 (17)	−0.0010 (13)	0.0017 (13)	0.0075 (14)
C1	0.0210 (19)	0.019 (2)	0.0261 (19)	−0.0004 (15)	0.0056 (15)	0.0076 (15)
C2	0.0232 (19)	0.030 (2)	0.029 (2)	−0.0013 (16)	0.0060 (16)	0.0045 (17)
C3	0.031 (2)	0.0197 (19)	0.029 (2)	−0.0054 (16)	0.0082 (16)	0.0019 (15)
C4	0.031 (2)	0.0190 (19)	0.033 (2)	−0.0001 (16)	0.0090 (17)	0.0038 (16)
C5	0.027 (2)	0.023 (2)	0.026 (2)	0.0029 (16)	0.0062 (16)	0.0034 (16)
C6	0.023 (2)	0.021 (2)	0.0204 (19)	−0.0013 (15)	0.0051 (15)	0.0023 (15)
C7	0.0219 (19)	0.025 (2)	0.0170 (17)	0.0017 (15)	0.0056 (15)	0.0024 (15)
C8	0.0211 (18)	0.0125 (17)	0.0278 (19)	−0.0021 (13)	0.0038 (15)	0.0057 (14)
C9	0.027 (2)	0.033 (2)	0.030 (2)	0.0033 (16)	0.0071 (16)	0.0091 (17)
C10	0.029 (2)	0.041 (2)	0.034 (2)	0.0030 (18)	0.0031 (17)	0.0135 (18)
C11	0.019 (2)	0.023 (2)	0.050 (3)	−0.0002 (16)	0.0029 (18)	0.0051 (19)
C12	0.022 (2)	0.026 (2)	0.039 (2)	−0.0034 (16)	0.0092 (17)	0.0000 (17)
C13	0.0233 (19)	0.026 (2)	0.0261 (19)	−0.0031 (15)	0.0026 (15)	0.0015 (15)
C14	0.0197 (17)	0.0169 (18)	0.0281 (19)	0.0003 (13)	0.0010 (14)	0.0061 (15)
C15	0.0255 (19)	0.0183 (18)	0.0276 (19)	0.0035 (14)	−0.0003 (15)	0.0079 (15)
C16	0.035 (2)	0.032 (2)	0.031 (2)	−0.0052 (18)	0.0058 (18)	0.0017 (17)
C17	0.053 (3)	0.043 (3)	0.033 (2)	0.002 (2)	0.013 (2)	0.002 (2)
C18	0.057 (3)	0.033 (2)	0.033 (2)	0.000 (2)	−0.003 (2)	−0.0015 (19)
C19	0.038 (2)	0.033 (2)	0.041 (2)	−0.0050 (18)	−0.008 (2)	0.0003 (19)
C20	0.027 (2)	0.030 (2)	0.037 (2)	−0.0005 (16)	0.0012 (17)	0.0049 (18)
C21	0.025 (2)	0.024 (2)	0.028 (2)	0.0019 (16)	0.0064 (16)	0.0076 (16)
C22	0.023 (2)	0.029 (2)	0.040 (2)	−0.0005 (16)	0.0058 (17)	0.0072 (17)
C23	0.027 (2)	0.034 (2)	0.034 (2)	0.0077 (17)	0.0035 (17)	0.0074 (18)
C24	0.032 (2)	0.025 (2)	0.033 (2)	0.0066 (16)	0.0078 (17)	0.0051 (17)
C25	0.033 (2)	0.0183 (19)	0.027 (2)	0.0001 (15)	0.0058 (16)	0.0014 (16)
C26	0.026 (2)	0.024 (2)	0.0185 (18)	0.0013 (16)	0.0027 (15)	0.0014 (15)
C27	0.027 (2)	0.024 (2)	0.0190 (19)	−0.0038 (17)	0.0024 (16)	0.0019 (16)
C28	0.0219 (19)	0.0190 (19)	0.030 (2)	0.0016 (14)	0.0045 (16)	0.0035 (15)
C29	0.047 (3)	0.033 (2)	0.028 (2)	−0.012 (2)	0.0061 (19)	0.0031 (18)
C30	0.057 (3)	0.035 (3)	0.045 (3)	−0.016 (2)	0.016 (2)	0.007 (2)
C31	0.034 (2)	0.028 (2)	0.055 (3)	−0.0101 (18)	0.016 (2)	−0.003 (2)
C32	0.027 (2)	0.031 (2)	0.046 (3)	−0.0041 (17)	0.0023 (18)	−0.0116 (19)
C33	0.028 (2)	0.032 (2)	0.033 (2)	−0.0008 (17)	0.0050 (17)	−0.0021 (17)
C34	0.0160 (18)	0.0213 (19)	0.034 (2)	0.0002 (14)	0.0019 (15)	0.0083 (16)
C35	0.0257 (19)	0.0245 (19)	0.029 (2)	−0.0012 (15)	0.0018 (16)	0.0118 (16)
C36	0.024 (2)	0.042 (2)	0.035 (2)	−0.0001 (17)	0.0027 (17)	0.0107 (19)
C37	0.040 (3)	0.064 (3)	0.030 (2)	−0.008 (2)	−0.0003 (19)	−0.003 (2)
C38	0.047 (3)	0.071 (3)	0.028 (2)	0.003 (2)	0.012 (2)	−0.001 (2)
C39	0.033 (2)	0.058 (3)	0.034 (2)	0.005 (2)	0.0096 (19)	0.005 (2)
C40	0.028 (2)	0.041 (2)	0.032 (2)	−0.0027 (18)	0.0067 (17)	0.0045 (18)
Ni2	0.0225 (2)	0.0252 (3)	0.0502 (3)	0.00671 (19)	0.0166 (2)	0.0165 (2)
O3	0.0309 (17)	0.0362 (19)	0.090 (3)	0.0120 (14)	0.0303 (17)	0.0344 (18)
O4	0.0270 (17)	0.0344 (18)	0.096 (3)	0.0105 (14)	0.0277 (17)	0.0312 (18)
N3	0.0229 (17)	0.0217 (17)	0.0297 (17)	0.0026 (14)	0.0094 (14)	0.0064 (14)
N4	0.0224 (16)	0.0213 (17)	0.0298 (17)	0.0037 (13)	0.0080 (13)	0.0068 (14)
C41	0.027 (2)	0.030 (2)	0.036 (2)	−0.0002 (16)	0.0120 (17)	0.0088 (17)
C42	0.029 (2)	0.035 (2)	0.042 (2)	0.0009 (17)	0.0146 (18)	0.0077 (19)
C43	0.033 (2)	0.039 (2)	0.040 (2)	−0.0084 (19)	0.0173 (19)	0.0040 (19)
C44	0.041 (2)	0.029 (2)	0.046 (3)	−0.0069 (19)	0.016 (2)	0.0057 (19)
C45	0.033 (2)	0.023 (2)	0.039 (2)	−0.0030 (16)	0.0100 (18)	0.0047 (17)
C46	0.029 (2)	0.028 (2)	0.023 (2)	−0.0018 (17)	0.0089 (16)	0.0011 (16)
C47	0.026 (2)	0.023 (2)	0.0203 (18)	−0.0007 (16)	0.0030 (15)	0.0008 (15)
C48	0.0254 (19)	0.0184 (19)	0.033 (2)	−0.0006 (15)	0.0072 (16)	0.0043 (16)
C49	0.040 (2)	0.020 (2)	0.031 (2)	0.0024 (17)	0.0017 (18)	0.0010 (17)
C50	0.042 (3)	0.025 (2)	0.054 (3)	0.0051 (19)	−0.012 (2)	0.001 (2)
C51	0.027 (2)	0.025 (2)	0.073 (3)	0.0041 (17)	0.002 (2)	−0.007 (2)
C52	0.034 (2)	0.025 (2)	0.055 (3)	0.0009 (17)	0.017 (2)	−0.0081 (19)
C53	0.030 (2)	0.026 (2)	0.032 (2)	−0.0007 (16)	0.0095 (17)	−0.0003 (16)
C54	0.0216 (18)	0.0197 (18)	0.0284 (19)	0.0027 (14)	0.0092 (15)	0.0075 (15)
C55	0.0269 (19)	0.0215 (19)	0.029 (2)	0.0041 (15)	0.0063 (16)	0.0093 (15)
C56	0.034 (2)	0.031 (2)	0.036 (2)	0.0003 (17)	0.0131 (18)	0.0053 (17)
C57	0.055 (3)	0.036 (2)	0.036 (2)	0.001 (2)	0.017 (2)	0.000 (2)
C58	0.062 (3)	0.045 (3)	0.036 (3)	−0.015 (2)	0.007 (2)	−0.002 (2)
C59	0.042 (3)	0.069 (4)	0.042 (3)	−0.021 (3)	0.006 (2)	0.006 (2)
C60	0.034 (2)	0.048 (3)	0.032 (2)	−0.0019 (19)	0.0102 (18)	0.0070 (19)
C61	0.025 (2)	0.027 (2)	0.042 (2)	0.0091 (17)	0.0096 (18)	0.0133 (18)
C62	0.026 (2)	0.036 (2)	0.044 (2)	0.0070 (17)	0.0118 (18)	0.0088 (19)
C63	0.030 (2)	0.027 (2)	0.043 (2)	0.0095 (17)	0.0040 (18)	−0.0022 (18)
C64	0.033 (2)	0.019 (2)	0.052 (3)	0.0008 (17)	0.004 (2)	0.0036 (18)
C65	0.027 (2)	0.022 (2)	0.040 (2)	0.0004 (16)	0.0070 (17)	−0.0008 (17)
C66	0.024 (2)	0.023 (2)	0.0234 (19)	0.0051 (16)	0.0045 (15)	0.0040 (16)
C67	0.0223 (19)	0.0221 (19)	0.0206 (18)	0.0020 (15)	0.0027 (14)	0.0023 (15)
C68	0.0220 (18)	0.0182 (18)	0.0245 (18)	0.0033 (14)	0.0062 (15)	0.0041 (14)
C69	0.027 (2)	0.036 (2)	0.027 (2)	−0.0041 (17)	0.0025 (16)	0.0038 (17)
C70	0.034 (2)	0.048 (3)	0.028 (2)	−0.0028 (19)	0.0102 (18)	0.0100 (19)
C71	0.025 (2)	0.028 (2)	0.038 (2)	0.0019 (17)	0.0117 (18)	0.0056 (18)
C72	0.022 (2)	0.031 (2)	0.032 (2)	−0.0011 (16)	0.0039 (16)	−0.0030 (17)
C73	0.027 (2)	0.031 (2)	0.0225 (19)	0.0025 (16)	0.0046 (15)	0.0024 (16)
C74	0.0195 (17)	0.0213 (18)	0.0284 (19)	0.0042 (14)	0.0078 (15)	0.0070 (15)
C75	0.031 (2)	0.0195 (19)	0.029 (2)	0.0014 (15)	0.0094 (16)	0.0088 (15)
C76	0.032 (2)	0.034 (2)	0.034 (2)	0.0052 (17)	0.0051 (18)	0.0056 (18)
C77	0.042 (3)	0.040 (3)	0.037 (2)	0.001 (2)	−0.0050 (19)	0.007 (2)
C78	0.061 (3)	0.029 (2)	0.032 (2)	−0.002 (2)	0.011 (2)	0.0014 (18)
C79	0.048 (3)	0.031 (2)	0.040 (2)	0.0052 (19)	0.019 (2)	0.0023 (19)
C80	0.033 (2)	0.030 (2)	0.034 (2)	0.0036 (17)	0.0124 (18)	0.0066 (17)
O5	0.0366 (18)	0.092 (3)	0.045 (2)	0.0128 (19)	0.0207 (16)	0.0217 (19)
C81	0.043 (3)	0.035 (2)	0.045 (3)	0.0078 (19)	0.015 (2)	0.012 (2)
C82	0.061 (3)	0.046 (3)	0.053 (3)	0.011 (2)	0.019 (3)	0.008 (2)
O6	0.042 (2)	0.092 (3)	0.058 (2)	−0.0029 (19)	0.0054 (17)	0.035 (2)
C83	0.049 (3)	0.068 (4)	0.088 (5)	0.008 (3)	0.019 (3)	0.024 (3)
C84	0.086 (4)	0.051 (3)	0.057 (3)	−0.003 (3)	0.021 (3)	0.010 (3)
O8	0.080 (3)	0.082 (3)	0.076 (3)	0.005 (3)	0.023 (3)	0.002 (3)
C87	0.109 (6)	0.099 (6)	0.067 (5)	−0.025 (5)	−0.009 (4)	0.026 (4)
C88	0.073 (11)	0.152 (17)	0.18 (2)	0.035 (11)	0.023 (13)	−0.023 (16)
C89	0.080 (18)	0.047 (11)	0.101 (16)	0.000 (9)	0.037 (12)	−0.003 (9)
O7	0.0346 (18)	0.083 (3)	0.045 (2)	0.0034 (18)	0.0019 (15)	0.0185 (18)
C85	0.041 (2)	0.038 (2)	0.043 (3)	−0.0007 (19)	0.012 (2)	0.011 (2)
C86	0.071 (4)	0.043 (3)	0.047 (3)	−0.005 (3)	0.002 (3)	0.004 (2)

### {2,2'-[(1S,2S)-1,2-Diphenylethane-1,2-diylbis(nitrilophenylmethanylylidene)]diphenolato}nickel(II) ethanol disolvate (1_EtOH) Geometric parameters (Å, º)

**Table:**

Ni1—O1	1.828 (3)	C45—C46	1.411 (6)
Ni1—O2	1.833 (3)	C46—C47	1.454 (6)
Ni1—N1	1.863 (3)	C47—C48	1.503 (5)
Ni1—N2	1.852 (3)	C48—C49	1.394 (6)
O1—C1	1.315 (5)	C48—C53	1.394 (5)
O2—C21	1.315 (5)	C49—H49	0.9500
N1—C7	1.298 (5)	C49—C50	1.385 (6)
N1—C14	1.478 (5)	C50—H50	0.9500
N2—C27	1.314 (5)	C50—C51	1.375 (7)
N2—C34	1.480 (5)	C51—H51	0.9500
C1—C2	1.407 (5)	C51—C52	1.378 (7)
C1—C6	1.420 (5)	C52—H52	0.9500
C2—H2	0.9500	C52—C53	1.387 (6)
C2—C3	1.380 (5)	C53—H53	0.9500
C3—H3	0.9500	C54—H54	1.0000
C3—C4	1.386 (6)	C54—C55	1.523 (5)
C4—H4	0.9500	C54—C74	1.540 (5)
C4—C5	1.368 (5)	C55—C56	1.379 (5)
C5—H5	0.9500	C55—C60	1.393 (6)
C5—C6	1.417 (5)	C56—H56	0.9500
C6—C7	1.458 (5)	C56—C57	1.393 (6)
C7—C8	1.499 (5)	C57—H57	0.9500
C8—C9	1.386 (5)	C57—C58	1.366 (7)
C8—C13	1.391 (5)	C58—H58	0.9500
C9—H9	0.9500	C58—C59	1.381 (7)
C9—C10	1.387 (5)	C59—H59	0.9500
C10—H10	0.9500	C59—C60	1.371 (6)
C10—C11	1.379 (6)	C60—H60	0.9500
C11—H11	0.9500	C61—C62	1.415 (5)
C11—C12	1.384 (6)	C61—C66	1.421 (6)
C12—H12	0.9500	C62—H62	0.9500
C12—C13	1.384 (5)	C62—C63	1.370 (6)
C13—H13	0.9500	C63—H63	0.9500
C14—H14	1.0000	C63—C64	1.403 (6)
C14—C15	1.520 (5)	C64—H64	0.9500
C14—C34	1.530 (5)	C64—C65	1.369 (6)
C15—C16	1.388 (5)	C65—H65	0.9500
C15—C20	1.387 (5)	C65—C66	1.411 (6)
C16—H16	0.9500	C66—C67	1.442 (5)
C16—C17	1.387 (6)	C67—C68	1.497 (5)
C17—H17	0.9500	C68—C69	1.389 (5)
C17—C18	1.372 (7)	C68—C73	1.393 (5)
C18—H18	0.9500	C69—H69	0.9500
C18—C19	1.375 (7)	C69—C70	1.385 (6)
C19—H19	0.9500	C70—H70	0.9500
C19—C20	1.394 (6)	C70—C71	1.378 (6)
C20—H20	0.9500	C71—H71	0.9500
C21—C22	1.412 (5)	C71—C72	1.381 (6)
C21—C26	1.412 (5)	C72—H72	0.9500
C22—H22	0.9500	C72—C73	1.375 (6)
C22—C23	1.375 (6)	C73—H73	0.9500
C23—H23	0.9500	C74—H74	1.0000
C23—C24	1.392 (6)	C74—C75	1.527 (5)
C24—H24	0.9500	C75—C76	1.381 (6)
C24—C25	1.387 (6)	C75—C80	1.400 (5)
C25—H25	0.9500	C76—H76	0.9500
C25—C26	1.414 (5)	C76—C77	1.390 (6)
C26—C27	1.450 (6)	C77—H77	0.9500
C27—C28	1.491 (5)	C77—C78	1.373 (6)
C28—C29	1.394 (6)	C78—H78	0.9500
C28—C33	1.385 (5)	C78—C79	1.380 (6)
C29—H29	0.9500	C79—H79	0.9500
C29—C30	1.378 (6)	C79—C80	1.386 (6)
C30—H30	0.9500	C80—H80	0.9500
C30—C31	1.378 (7)	O5—H5A	0.8400
C31—H31	0.9500	O5—C81	1.410 (5)
C31—C32	1.368 (6)	C81—H81A	0.9900
C32—H32	0.9500	C81—H81B	0.9900
C32—C33	1.394 (6)	C81—C82	1.502 (6)
C33—H33	0.9500	C82—H82A	0.9800
C34—H34	1.0000	C82—H82B	0.9800
C34—C35	1.518 (5)	C82—H82C	0.9800
C35—C36	1.390 (5)	O6—H6	0.8400
C35—C40	1.390 (6)	O6—C83	1.460 (7)
C36—H36	0.9500	C83—H83A	0.9900
C36—C37	1.378 (6)	C83—H83B	0.9900
C37—H37	0.9500	C83—C84	1.449 (8)
C37—C38	1.370 (7)	C84—H84A	0.9800
C38—H38	0.9500	C84—H84B	0.9800
C38—C39	1.378 (7)	C84—H84C	0.9800
C39—H39	0.9500	O8—H8	0.8400
C39—C40	1.370 (6)	O8—C87	1.275 (8)
C40—H40	0.9500	C87—H87A	0.9900
Ni2—O3	1.838 (3)	C87—H87B	0.9900
Ni2—O4	1.826 (3)	C87—H87C	0.9900
Ni2—N3	1.852 (3)	C87—H87D	0.9900
Ni2—N4	1.860 (3)	C87—C88	1.293 (17)
O3—C41	1.300 (5)	C87—C89	1.50 (2)
O4—C61	1.307 (5)	C88—H88A	0.9800
N3—C47	1.308 (5)	C88—H88B	0.9800
N3—C54	1.478 (5)	C88—H88C	0.9800
N4—C67	1.300 (5)	C89—H89A	0.9800
N4—C74	1.472 (5)	C89—H89B	0.9800
C41—C42	1.423 (6)	C89—H89C	0.9800
C41—C46	1.413 (6)	O7—H7	0.8400
C42—H42	0.9500	O7—C85	1.418 (5)
C42—C43	1.358 (6)	C85—H85A	0.9900
C43—H43	0.9500	C85—H85B	0.9900
C43—C44	1.383 (6)	C85—C86	1.496 (7)
C44—H44	0.9500	C86—H86A	0.9800
C44—C45	1.378 (6)	C86—H86B	0.9800
C45—H45	0.9500	C86—H86C	0.9800
			
O1—Ni1—O2	83.98 (12)	N3—C47—C46	122.6 (4)
O1—Ni1—N1	94.47 (13)	N3—C47—C48	119.1 (3)
O1—Ni1—N2	176.84 (15)	C46—C47—C48	118.3 (3)
O2—Ni1—N1	178.39 (14)	C49—C48—C47	120.2 (3)
O2—Ni1—N2	94.11 (13)	C49—C48—C53	120.6 (4)
N2—Ni1—N1	87.42 (14)	C53—C48—C47	119.1 (3)
C1—O1—Ni1	127.4 (2)	C48—C49—H49	120.5
C21—O2—Ni1	127.3 (3)	C50—C49—C48	119.0 (4)
C7—N1—Ni1	127.8 (3)	C50—C49—H49	120.5
C7—N1—C14	120.3 (3)	C49—C50—H50	119.6
C14—N1—Ni1	111.7 (2)	C51—C50—C49	120.7 (4)
C27—N2—Ni1	128.5 (3)	C51—C50—H50	119.6
C27—N2—C34	121.1 (3)	C50—C51—H51	120.0
C34—N2—Ni1	110.4 (2)	C50—C51—C52	120.1 (4)
O1—C1—C2	117.0 (3)	C52—C51—H51	120.0
O1—C1—C6	124.5 (3)	C51—C52—H52	119.6
C2—C1—C6	118.5 (3)	C51—C52—C53	120.8 (4)
C1—C2—H2	119.5	C53—C52—H52	119.6
C3—C2—C1	121.1 (4)	C48—C53—H53	120.6
C3—C2—H2	119.5	C52—C53—C48	118.8 (4)
C2—C3—H3	119.7	C52—C53—H53	120.6
C2—C3—C4	120.6 (4)	N3—C54—H54	109.2
C4—C3—H3	119.7	N3—C54—C55	112.2 (3)
C3—C4—H4	120.2	N3—C54—C74	105.1 (3)
C5—C4—C3	119.7 (4)	C55—C54—H54	109.2
C5—C4—H4	120.2	C55—C54—C74	112.0 (3)
C4—C5—H5	119.2	C74—C54—H54	109.2
C4—C5—C6	121.6 (4)	C56—C55—C54	123.5 (3)
C6—C5—H5	119.2	C56—C55—C60	118.7 (4)
C1—C6—C7	121.5 (3)	C60—C55—C54	117.8 (3)
C5—C6—C1	118.4 (3)	C55—C56—H56	119.7
C5—C6—C7	119.9 (3)	C55—C56—C57	120.6 (4)
N1—C7—C6	122.5 (3)	C57—C56—H56	119.7
N1—C7—C8	120.8 (3)	C56—C57—H57	119.9
C6—C7—C8	116.6 (3)	C58—C57—C56	120.2 (4)
C9—C8—C7	121.0 (3)	C58—C57—H57	119.9
C9—C8—C13	120.0 (3)	C57—C58—H58	120.3
C13—C8—C7	118.8 (3)	C57—C58—C59	119.4 (4)
C8—C9—H9	120.2	C59—C58—H58	120.3
C8—C9—C10	119.6 (4)	C58—C59—H59	119.5
C10—C9—H9	120.2	C60—C59—C58	120.9 (4)
C9—C10—H10	119.8	C60—C59—H59	119.5
C11—C10—C9	120.3 (4)	C55—C60—H60	119.9
C11—C10—H10	119.8	C59—C60—C55	120.2 (4)
C10—C11—H11	119.9	C59—C60—H60	119.9
C10—C11—C12	120.3 (4)	O4—C61—C62	117.2 (4)
C12—C11—H11	119.9	O4—C61—C66	124.4 (4)
C11—C12—H12	120.1	C62—C61—C66	118.4 (4)
C11—C12—C13	119.8 (4)	C61—C62—H62	119.3
C13—C12—H12	120.1	C63—C62—C61	121.4 (4)
C8—C13—H13	120.0	C63—C62—H62	119.3
C12—C13—C8	120.1 (4)	C62—C63—H63	119.7
C12—C13—H13	120.0	C62—C63—C64	120.7 (4)
N1—C14—H14	109.2	C64—C63—H63	119.7
N1—C14—C15	113.0 (3)	C63—C64—H64	120.6
N1—C14—C34	104.3 (3)	C65—C64—C63	118.7 (4)
C15—C14—H14	109.2	C65—C64—H64	120.6
C15—C14—C34	111.9 (3)	C64—C65—H65	118.7
C34—C14—H14	109.2	C64—C65—C66	122.7 (4)
C16—C15—C14	123.7 (3)	C66—C65—H65	118.7
C20—C15—C14	117.5 (3)	C61—C66—C67	121.3 (4)
C20—C15—C16	118.8 (4)	C65—C66—C61	118.1 (4)
C15—C16—H16	119.9	C65—C66—C67	120.5 (4)
C17—C16—C15	120.2 (4)	N4—C67—C66	122.9 (3)
C17—C16—H16	119.9	N4—C67—C68	120.3 (3)
C16—C17—H17	119.6	C66—C67—C68	116.9 (3)
C18—C17—C16	120.9 (4)	C69—C68—C67	121.3 (3)
C18—C17—H17	119.6	C69—C68—C73	118.8 (3)
C17—C18—H18	120.2	C73—C68—C67	119.7 (3)
C17—C18—C19	119.6 (4)	C68—C69—H69	119.9
C19—C18—H18	120.2	C70—C69—C68	120.2 (4)
C18—C19—H19	119.9	C70—C69—H69	119.9
C18—C19—C20	120.1 (4)	C69—C70—H70	119.7
C20—C19—H19	119.9	C71—C70—C69	120.5 (4)
C15—C20—C19	120.5 (4)	C71—C70—H70	119.7
C15—C20—H20	119.8	C70—C71—H71	120.3
C19—C20—H20	119.8	C70—C71—C72	119.4 (4)
O2—C21—C22	116.9 (3)	C72—C71—H71	120.3
O2—C21—C26	124.2 (3)	C71—C72—H72	119.7
C26—C21—C22	118.8 (4)	C73—C72—C71	120.5 (4)
C21—C22—H22	119.5	C73—C72—H72	119.7
C23—C22—C21	121.0 (4)	C68—C73—H73	119.8
C23—C22—H22	119.5	C72—C73—C68	120.5 (4)
C22—C23—H23	119.6	C72—C73—H73	119.8
C22—C23—C24	120.9 (4)	N4—C74—C54	104.9 (3)
C24—C23—H23	119.6	N4—C74—H74	109.1
C23—C24—H24	120.5	N4—C74—C75	113.0 (3)
C25—C24—C23	119.0 (4)	C54—C74—H74	109.1
C25—C24—H24	120.5	C75—C74—C54	111.4 (3)
C24—C25—H25	119.2	C75—C74—H74	109.1
C24—C25—C26	121.5 (4)	C76—C75—C74	124.0 (3)
C26—C25—H25	119.2	C76—C75—C80	118.4 (4)
C21—C26—C25	118.6 (4)	C80—C75—C74	117.6 (3)
C21—C26—C27	122.4 (4)	C75—C76—H76	119.7
C25—C26—C27	118.9 (4)	C75—C76—C77	120.6 (4)
N2—C27—C26	121.4 (4)	C77—C76—H76	119.7
N2—C27—C28	119.1 (4)	C76—C77—H77	119.7
C26—C27—C28	119.5 (3)	C78—C77—C76	120.6 (4)
C29—C28—C27	121.5 (3)	C78—C77—H77	119.7
C33—C28—C27	119.6 (3)	C77—C78—H78	120.1
C33—C28—C29	118.9 (4)	C77—C78—C79	119.8 (4)
C28—C29—H29	119.8	C79—C78—H78	120.1
C30—C29—C28	120.3 (4)	C78—C79—H79	120.1
C30—C29—H29	119.8	C78—C79—C80	119.9 (4)
C29—C30—H30	119.7	C80—C79—H79	120.1
C29—C30—C31	120.7 (4)	C75—C80—H80	119.6
C31—C30—H30	119.7	C79—C80—C75	120.8 (4)
C30—C31—H31	120.3	C79—C80—H80	119.6
C32—C31—C30	119.4 (4)	C81—O5—H5A	109.5
C32—C31—H31	120.3	O5—C81—H81A	109.2
C31—C32—H32	119.6	O5—C81—H81B	109.2
C31—C32—C33	120.8 (4)	O5—C81—C82	112.2 (4)
C33—C32—H32	119.6	H81A—C81—H81B	107.9
C28—C33—C32	119.9 (4)	C82—C81—H81A	109.2
C28—C33—H33	120.1	C82—C81—H81B	109.2
C32—C33—H33	120.1	C81—C82—H82A	109.5
N2—C34—C14	105.7 (3)	C81—C82—H82B	109.5
N2—C34—H34	109.7	C81—C82—H82C	109.5
N2—C34—C35	112.0 (3)	H82A—C82—H82B	109.5
C14—C34—H34	109.7	H82A—C82—H82C	109.5
C35—C34—C14	109.9 (3)	H82B—C82—H82C	109.5
C35—C34—H34	109.7	C83—O6—H6	109.5
C36—C35—C34	123.5 (3)	O6—C83—H83A	109.5
C36—C35—C40	117.9 (4)	O6—C83—H83B	109.5
C40—C35—C34	118.4 (3)	H83A—C83—H83B	108.1
C35—C36—H36	120.0	C84—C83—O6	110.7 (5)
C37—C36—C35	120.0 (4)	C84—C83—H83A	109.5
C37—C36—H36	120.0	C84—C83—H83B	109.5
C36—C37—H37	119.4	C83—C84—H84A	109.5
C38—C37—C36	121.3 (4)	C83—C84—H84B	109.5
C38—C37—H37	119.4	C83—C84—H84C	109.5
C37—C38—H38	120.3	H84A—C84—H84B	109.5
C37—C38—C39	119.4 (4)	H84A—C84—H84C	109.5
C39—C38—H38	120.3	H84B—C84—H84C	109.5
C38—C39—H39	120.2	C87—O8—H8	109.5
C40—C39—C38	119.6 (4)	O8—C87—H87A	105.2
C40—C39—H39	120.2	O8—C87—H87B	105.2
C35—C40—H40	119.1	O8—C87—H87C	107.0
C39—C40—C35	121.8 (4)	O8—C87—H87D	107.0
C39—C40—H40	119.1	O8—C87—C88	128.4 (11)
O3—Ni2—N3	94.70 (13)	O8—C87—C89	121.4 (11)
O3—Ni2—N4	176.86 (16)	H87A—C87—H87B	105.9
O4—Ni2—O3	83.89 (13)	H87C—C87—H87D	106.7
O4—Ni2—N3	177.94 (16)	C88—C87—H87A	105.2
O4—Ni2—N4	93.95 (13)	C88—C87—H87B	105.2
N3—Ni2—N4	87.52 (14)	C89—C87—H87C	107.0
C41—O3—Ni2	127.9 (3)	C89—C87—H87D	107.0
C61—O4—Ni2	127.1 (3)	C87—C88—H88A	109.5
C47—N3—Ni2	127.8 (3)	C87—C88—H88B	109.5
C47—N3—C54	120.8 (3)	C87—C88—H88C	109.5
C54—N3—Ni2	111.4 (2)	H88A—C88—H88B	109.5
C67—N4—Ni2	127.8 (3)	H88A—C88—H88C	109.5
C67—N4—C74	121.1 (3)	H88B—C88—H88C	109.5
C74—N4—Ni2	111.1 (2)	C87—C89—H89A	109.5
O3—C41—C42	117.2 (4)	C87—C89—H89B	109.5
O3—C41—C46	124.4 (4)	C87—C89—H89C	109.5
C46—C41—C42	118.4 (4)	H89A—C89—H89B	109.5
C41—C42—H42	119.5	H89A—C89—H89C	109.5
C43—C42—C41	120.9 (4)	H89B—C89—H89C	109.5
C43—C42—H42	119.5	C85—O7—H7	109.5
C42—C43—H43	119.4	O7—C85—H85A	109.3
C42—C43—C44	121.1 (4)	O7—C85—H85B	109.3
C44—C43—H43	119.4	O7—C85—C86	111.6 (4)
C43—C44—H44	120.2	H85A—C85—H85B	108.0
C45—C44—C43	119.5 (4)	C86—C85—H85A	109.3
C45—C44—H44	120.2	C86—C85—H85B	109.3
C44—C45—H45	119.3	C85—C86—H86A	109.5
C44—C45—C46	121.3 (4)	C85—C86—H86B	109.5
C46—C45—H45	119.3	C85—C86—H86C	109.5
C41—C46—C47	121.9 (4)	H86A—C86—H86B	109.5
C45—C46—C41	118.6 (4)	H86A—C86—H86C	109.5
C45—C46—C47	119.4 (4)	H86B—C86—H86C	109.5
			
Ni1—O1—C1—C2	177.0 (3)	Ni2—O3—C41—C42	179.8 (3)
Ni1—O1—C1—C6	−3.1 (6)	Ni2—O3—C41—C46	0.4 (7)
Ni1—O2—C21—C22	175.4 (3)	Ni2—O4—C61—C62	168.8 (3)
Ni1—O2—C21—C26	−5.3 (6)	Ni2—O4—C61—C66	−12.6 (7)
Ni1—N1—C7—C6	10.3 (5)	Ni2—N3—C47—C46	6.8 (5)
Ni1—N1—C7—C8	−168.9 (3)	Ni2—N3—C47—C48	−173.3 (3)
Ni1—N1—C14—C15	−85.4 (3)	Ni2—N3—C54—C55	−85.0 (3)
Ni1—N1—C14—C34	36.3 (3)	Ni2—N3—C54—C74	36.9 (3)
Ni1—N2—C27—C26	8.2 (5)	Ni2—N4—C67—C66	5.9 (5)
Ni1—N2—C27—C28	−173.9 (3)	Ni2—N4—C67—C68	−174.3 (3)
Ni1—N2—C34—C14	39.5 (3)	Ni2—N4—C74—C54	38.2 (3)
Ni1—N2—C34—C35	−80.1 (3)	Ni2—N4—C74—C75	−83.4 (3)
O1—Ni1—O2—C21	−169.3 (3)	O3—Ni2—O4—C61	−164.7 (4)
O1—Ni1—N1—C7	−14.5 (3)	O3—Ni2—N3—C47	−8.7 (4)
O1—Ni1—N1—C14	169.9 (2)	O3—Ni2—N3—C54	168.7 (3)
O1—C1—C2—C3	179.8 (4)	O3—C41—C42—C43	−178.3 (4)
O1—C1—C6—C5	178.5 (4)	O3—C41—C46—C45	177.7 (4)
O1—C1—C6—C7	−5.7 (6)	O3—C41—C46—C47	−4.9 (7)
O2—Ni1—O1—C1	−168.8 (3)	O4—Ni2—O3—C41	−176.3 (4)
O2—Ni1—N2—C27	−14.8 (4)	O4—Ni2—N4—C67	−14.4 (3)
O2—Ni1—N2—C34	163.5 (2)	O4—Ni2—N4—C74	166.3 (3)
O2—C21—C22—C23	−177.3 (4)	O4—C61—C62—C63	177.6 (4)
O2—C21—C26—C25	176.9 (4)	O4—C61—C66—C65	−178.2 (4)
O2—C21—C26—C27	−6.4 (6)	O4—C61—C66—C67	−1.6 (7)
N1—Ni1—O1—C1	10.8 (3)	N3—Ni2—O3—C41	5.2 (4)
N1—Ni1—N2—C27	165.7 (3)	N3—Ni2—N4—C67	164.2 (3)
N1—Ni1—N2—C34	−16.0 (2)	N3—Ni2—N4—C74	−15.2 (3)
N1—C14—C15—C16	12.3 (5)	N3—C54—C55—C56	−0.5 (5)
N1—C14—C15—C20	−168.5 (3)	N3—C54—C55—C60	179.6 (3)
N1—C14—C34—N2	−47.4 (4)	N3—C54—C74—N4	−46.9 (3)
N1—C14—C34—C35	73.7 (3)	N3—C54—C74—C75	75.7 (3)
N1—C7—C8—C9	−101.1 (4)	N3—C47—C48—C49	−99.9 (5)
N1—C7—C8—C13	84.1 (4)	N3—C47—C48—C53	79.2 (5)
N2—Ni1—O2—C21	13.2 (3)	N4—Ni2—O4—C61	17.6 (4)
N2—Ni1—N1—C7	162.9 (3)	N4—Ni2—N3—C47	169.0 (3)
N2—Ni1—N1—C14	−12.6 (3)	N4—Ni2—N3—C54	−13.5 (2)
N2—C27—C28—C29	−97.7 (5)	N4—C67—C68—C69	−98.7 (4)
N2—C27—C28—C33	80.3 (5)	N4—C67—C68—C73	85.4 (4)
N2—C34—C35—C36	17.7 (5)	N4—C74—C75—C76	11.4 (5)
N2—C34—C35—C40	−168.5 (3)	N4—C74—C75—C80	−170.1 (3)
C1—C2—C3—C4	1.6 (6)	C41—C42—C43—C44	−0.1 (7)
C1—C6—C7—N1	1.9 (6)	C41—C46—C47—N3	1.2 (6)
C1—C6—C7—C8	−178.8 (3)	C41—C46—C47—C48	−178.6 (4)
C2—C1—C6—C5	−1.6 (5)	C42—C41—C46—C45	−1.7 (6)
C2—C1—C6—C7	174.2 (3)	C42—C41—C46—C47	175.6 (4)
C2—C3—C4—C5	−1.3 (6)	C42—C43—C44—C45	−0.3 (7)
C3—C4—C5—C6	−0.5 (6)	C43—C44—C45—C46	−0.3 (7)
C4—C5—C6—C1	1.9 (5)	C44—C45—C46—C41	1.3 (6)
C4—C5—C6—C7	−173.9 (3)	C44—C45—C46—C47	−176.1 (4)
C5—C6—C7—N1	177.6 (3)	C45—C46—C47—N3	178.6 (4)
C5—C6—C7—C8	−3.1 (5)	C45—C46—C47—C48	−1.3 (5)
C6—C1—C2—C3	−0.2 (6)	C46—C41—C42—C43	1.2 (6)
C6—C7—C8—C9	79.6 (4)	C46—C47—C48—C49	80.0 (5)
C6—C7—C8—C13	−95.2 (4)	C46—C47—C48—C53	−100.9 (4)
C7—N1—C14—C15	98.7 (4)	C47—N3—C54—C55	92.7 (4)
C7—N1—C14—C34	−139.6 (3)	C47—N3—C54—C74	−145.4 (3)
C7—C8—C9—C10	−173.3 (3)	C47—C48—C49—C50	179.0 (4)
C7—C8—C13—C12	174.2 (3)	C47—C48—C53—C52	−178.7 (4)
C8—C9—C10—C11	−0.7 (6)	C48—C49—C50—C51	−0.6 (7)
C9—C8—C13—C12	−0.7 (6)	C49—C48—C53—C52	0.4 (6)
C9—C10—C11—C12	−0.8 (6)	C49—C50—C51—C52	1.1 (7)
C10—C11—C12—C13	1.6 (6)	C50—C51—C52—C53	−0.8 (7)
C11—C12—C13—C8	−0.9 (6)	C51—C52—C53—C48	0.1 (6)
C13—C8—C9—C10	1.4 (6)	C53—C48—C49—C50	−0.1 (6)
C14—N1—C7—C6	−174.5 (3)	C54—N3—C47—C46	−170.5 (3)
C14—N1—C7—C8	6.3 (5)	C54—N3—C47—C48	9.4 (5)
C14—C15—C16—C17	178.9 (4)	C54—C55—C56—C57	179.2 (4)
C14—C15—C20—C19	−178.8 (3)	C54—C55—C60—C59	−178.5 (4)
C14—C34—C35—C36	−99.5 (4)	C54—C74—C75—C76	−106.4 (4)
C14—C34—C35—C40	74.3 (4)	C54—C74—C75—C80	72.1 (4)
C15—C14—C34—N2	75.1 (4)	C55—C54—C74—N4	75.2 (4)
C15—C14—C34—C35	−163.8 (3)	C55—C54—C74—C75	−162.2 (3)
C15—C16—C17—C18	0.1 (7)	C55—C56—C57—C58	0.1 (7)
C16—C15—C20—C19	0.4 (6)	C56—C55—C60—C59	1.6 (7)
C16—C17—C18—C19	0.2 (7)	C56—C57—C58—C59	0.1 (8)
C17—C18—C19—C20	−0.1 (7)	C57—C58—C59—C60	0.6 (8)
C18—C19—C20—C15	−0.2 (6)	C58—C59—C60—C55	−1.4 (8)
C20—C15—C16—C17	−0.4 (6)	C60—C55—C56—C57	−0.9 (6)
C21—C22—C23—C24	−0.7 (6)	C61—C62—C63—C64	0.4 (7)
C21—C26—C27—N2	4.9 (6)	C61—C66—C67—N4	5.0 (6)
C21—C26—C27—C28	−173.0 (3)	C61—C66—C67—C68	−174.8 (3)
C22—C21—C26—C25	−3.9 (5)	C62—C61—C66—C65	0.4 (6)
C22—C21—C26—C27	172.8 (4)	C62—C61—C66—C67	176.9 (4)
C22—C23—C24—C25	−1.6 (6)	C62—C63—C64—C65	1.0 (6)
C23—C24—C25—C26	1.1 (6)	C63—C64—C65—C66	−1.7 (6)
C24—C25—C26—C21	1.7 (6)	C64—C65—C66—C61	1.0 (6)
C24—C25—C26—C27	−175.1 (3)	C64—C65—C66—C67	−175.6 (4)
C25—C26—C27—N2	−178.4 (4)	C65—C66—C67—N4	−178.5 (4)
C25—C26—C27—C28	3.6 (5)	C65—C66—C67—C68	1.6 (5)
C26—C21—C22—C23	3.4 (6)	C66—C61—C62—C63	−1.0 (7)
C26—C27—C28—C29	80.3 (5)	C66—C67—C68—C69	81.1 (5)
C26—C27—C28—C33	−101.7 (4)	C66—C67—C68—C73	−94.8 (4)
C27—N2—C34—C14	−142.1 (3)	C67—N4—C74—C54	−141.2 (3)
C27—N2—C34—C35	98.3 (4)	C67—N4—C74—C75	97.2 (4)
C27—C28—C29—C30	176.7 (4)	C67—C68—C69—C70	−174.7 (4)
C27—C28—C33—C32	−178.1 (4)	C67—C68—C73—C72	175.1 (3)
C28—C29—C30—C31	0.6 (7)	C68—C69—C70—C71	−0.3 (6)
C29—C28—C33—C32	0.0 (6)	C69—C68—C73—C72	−0.9 (6)
C29—C30—C31—C32	1.6 (7)	C69—C70—C71—C72	−0.8 (7)
C30—C31—C32—C33	−3.0 (7)	C70—C71—C72—C73	1.1 (6)
C31—C32—C33—C28	2.2 (6)	C71—C72—C73—C68	−0.2 (6)
C33—C28—C29—C30	−1.3 (6)	C73—C68—C69—C70	1.2 (6)
C34—N2—C27—C26	−169.9 (3)	C74—N4—C67—C66	−174.9 (3)
C34—N2—C27—C28	8.1 (5)	C74—N4—C67—C68	5.0 (5)
C34—C14—C15—C16	−105.1 (4)	C74—C54—C55—C56	−118.4 (4)
C34—C14—C15—C20	74.1 (4)	C74—C54—C55—C60	61.7 (4)
C34—C35—C36—C37	174.1 (4)	C74—C75—C76—C77	178.4 (4)
C34—C35—C40—C39	−174.3 (4)	C74—C75—C80—C79	−178.5 (4)
C35—C36—C37—C38	0.4 (7)	C75—C76—C77—C78	0.2 (7)
C36—C35—C40—C39	−0.2 (6)	C76—C75—C80—C79	0.1 (6)
C36—C37—C38—C39	−1.2 (8)	C76—C77—C78—C79	−0.2 (7)
C37—C38—C39—C40	1.3 (8)	C77—C78—C79—C80	0.1 (7)
C38—C39—C40—C35	−0.6 (7)	C78—C79—C80—C75	−0.1 (6)
C40—C35—C36—C37	0.3 (6)	C80—C75—C76—C77	−0.1 (6)

### {2,2'-[(1S,2S)-1,2-Diphenylethane-1,2-diylbis(nitrilophenylmethanylylidene)]diphenolato}nickel(II) ethanol disolvate (1_EtOH) Hydrogen-bond geometry (Å, º)

**Table:**

*D*—H···*A*	*D*—H	H···*A*	*D*···*A*	*D*—H···*A*
O5—H5*A*···O3	0.84	2.17	2.964 (5)	156
O6—H6···O1	0.84	2.13	2.928 (5)	158
O8—H8···O6	0.84	1.92	2.760 (6)	174
O7—H7···O2	0.84	2.16	2.988 (4)	170
C60—H60···O5^i^	0.95	2.38	3.312 (6)	166
C12—H12···O8^i^	0.95	2.44	3.388 (7)	175
C40—H40···O7^i^	0.95	2.48	3.328 (5)	148

Symmetry code: (i) *x*−1, *y*, *z*.
